# Ethnic differences in total and HDL cholesterol among Turkish, Moroccan and Dutch ethnic groups living in Amsterdam, the Netherlands

**DOI:** 10.1186/1471-2458-10-740

**Published:** 2010-11-30

**Authors:** Joanne K Ujcic-Voortman, Griët Bos, Caroline A Baan, Daan G Uitenbroek, Arnoud P Verhoeff, Jacob C Seidell

**Affiliations:** 1 Public Health Service Amsterdam, Department of Epidemiology, Documentation and Health Promotion, Amsterdam, the Netherlands; 2 National Institute of Public Health and the Environment, Centre for Prevention and Health Services Research, Bilthoven, the Netherlands; 3 University of Amsterdam, Faculty of Social and Behavioural Sciences, Amsterdam, the Netherlands; 4 VU University Amsterdam, Faculty of Earth and Life Sciences, Amsterdam, the Netherlands

## Abstract

**Background:**

High total cholesterol and low HDL (high-density lipoprotein) cholesterol are important determinants of cardiovascular disease. Little is known about dyslipidemia among Turkish and Moroccan migrants, two of the largest ethnic minority groups in several European countries. This study examines ethnic differences in total and HDL cholesterol levels between Dutch, Turkish and Moroccan ethnic groups.

**Methods:**

Data were collected in the setting of a general health survey, in Amsterdam, the Netherlands, in 2004. Total response rate was 45% (Dutch: 46%, Turks: 50%, Moroccans: 39%). From 1,220 adults information on history of hypercholesterolemia, lifestyle and demographic background was obtained via health interviews. In a physical examination measurements of anthropometry and blood pressure were performed and blood was collected. Total and HDL cholesterol were determined in serum.

**Results:**

Total cholesterol levels were lower and hypercholesterolemia was less prevalent among the Moroccan and Turkish than the Dutch ethnic population. HDL cholesterol was also relatively low among these migrant groups. The resulting total/HDL cholesterol ratio was particularly unfavourable among the Turkish ethnic group. Controlling for Body Mass Index and alcohol abstinence substantially attenuated ethnic differences in HDL cholesterol levels and total/HDL cholesterol ratio.

**Conclusions:**

Total cholesterol levels are relatively low in Turkish and Moroccan migrants. However part of this advantage is off-set by their relatively low levels of HDL cholesterol, resulting in an unfavourable total/HDL cholesterol ratio, particularly in the Turkish population. Important factors in explaining ethnic differences are the relatively high Body Mass Index and level of alcohol abstinence in these migrant groups.

## Background

Cardiovascular disease (CVD) is the leading cause of death worldwide and is a major contributor to the global burden of disease [1]. Smoking, high (systolic) blood pressure, diabetes and obesity are important determinants of CVD as is high cholesterol, more specifically high LDL (low density lipoprotein) cholesterol, low HDL (high density lipoprotein) cholesterol and a high total/HDL cholesterol ratio [2-5]. In fact, high blood pressure and high cholesterol account for 46% and 24% of risk of cardiovascular disease mortality worldwide, respectively [1].

In a previous health interview survey in Amsterdam, the Netherlands, Turkish and Moroccan migrants were identified as vulnerable groups with poorer health perspectives compared to their Dutch counterparts. Diabetes and obesity, both strongly associated with cardiovascular risk, are more prevalent among these groups [6,7]. Prevalence rates of CVD are found to be relatively high among Turkish migrants, and risk of death from cerebrovascular accidents is relatively high among Turkish migrant men [6,8]. However, compared to the Dutch, risk of coronary heart disease mortality is lower among Turkish migrant women and risk of death from all CVD is lower among Moroccan migrant men [8].

Despite differences in morbidity and mortality there is still limited information concerning major risk factors for CVD among Turkish and Moroccan migrant groups [9]. There is a particular lack of objective data based on physical examination to back up self-reported survey data. Gathering more information on cardiovascular disease risk among these groups is of great importance as migrants from Turkey and Morocco are among the largest and still growing ethnic minority groups in several European countries [9]. In Amsterdam, Turkish and Moroccan migrants account for around 5% and 9% of the total population and their numbers are expected to rise in the coming years due to their relatively high birth and immigration rates [6,10]. However, in many epidemiological studies they are poorly represented and little is known about the prevalence of hypercholesterolemia and dyslipidemia among these migrant groups.

In this study, we investigated serum levels of total cholesterol, HDL cholesterol and the total/HDL cholesterol ratio among Turkish, Moroccan and Dutch ethnic groups in the Amsterdam population. The main objective was to determine whether there are ethnic differences in serum total and HDL cholesterol levels between these groups. More insight into possible explanations for ethnic differences in cholesterol levels can help to better target ethnic differences in dyslipidemia. Therefore, our second aim was to examine whether these ethnic differences can be explained by other known determinants of dyslipidemia and cardiovascular disease risk, such as socioeconomic status (SES), Body Mass Index (BMI), diabetes, smoking, alcohol consumption and physical activity [2-5,11].

## Methods

In 2004 a general health survey, combining a health interview and physical examination, was conducted by the Public Health Service of Amsterdam in collaboration with the National Institute of Public Health and the Environment. The data obtained from this cross-sectional survey in the Amsterdam population were used for this study [12,13].

### Participants

The study sample was drawn from the municipal population register within five districts in Amsterdam, which combined were representative of the total Amsterdam population. To ensure sufficient numbers in each age and ethnic group, the sample was stratified by ethnicity (Dutch, Turkish, Moroccan) and five age groups (18-34 years, 35-44 years, 45-54 years, 55-64 years, 65 years and older). Per stratum a random sample was drawn. Selected residents of the five districts were invited to participate in a health interview and a health examination at a local childcare centre. All participants signed an informed consent form. The study protocol was approved by the Medical Ethical Committee of the Academic Medical Centre, University of Amsterdam.

### Health interview

A health interview was conducted in the respondent's language of preference (Dutch, Turkish, Moroccan-Arabic, Berber or English). Arabic, Turkish and English translations of the questionnaire, done by certified interpreters, were available (for the English translation of the questionnaire, see Additional file 1). The interview covered several aspects of physical and psychological health, lifestyle and demographic factors, including history of hypercholesterolemia, current smoking, alcohol consumption and physical activity (SQUASH questionnaire [14]). Physically active was defined as at least half an hour of moderate activity on at least five days a week. As a proxy for SES self-reported educational level was used, divided into three categories: low (up to primary school), medium (up to secondary school) and high (higher professional education or university). Ethnicity was based on the self reported country of birth of the respondent and his/her parents. If one of these was Turkey or Morocco, the respondent was considered to be Turkish or Moroccan, respectively. Dutch means that both of the respondent's parents were born in the Netherlands.

### Health examination

A health examination was conducted by a trained nurse. Body height and weight were measured with respondents wearing light indoor clothing without shoes, using a wall mounted stadiometer and a calibrated analogue scale, respectively. Body weight was adjusted for clothing weight by subtracting 1 kg. Blood samples were collected (non-fasting) and total and HDL cholesterol, glucose and HbA_1c _(glycosylated haemoglobin) were determined in serum using standard laboratory techniques (Hitachi 911, Roche Diagnostics, Mannheim). Hypercholesterolemia was defined as self-reported elevated cholesterol levels and current use of lipid lowering medication and/or elevated serum total cholesterol levels: 6.5 mmol/l or higher, in accordance with Dutch guidelines for cardiovascular risk management [15]. HDL cholesterol levels lower than 1.0 mmol/l in men and 1.2 mmol/l in women were defined as low [16]. A total/HDL cholesterol ratio of greater than 5 was considered to be high [17]. Diabetes was defined as self-reported diabetes and current use of anti-diabetic medication or non-fasting glucose levels above 11.0 mmol/l with HbA_1c _levels above 48 mmol/mol (6.5%). To ensure correctness of data-entry all entered questionnaire and health examination data were double-checked with the original forms.

### Statistical analyses

Respondents who didn't participate in the physical examination (n = 8) or from whom no or insufficient amounts of blood were collected (n = 101) were excluded from analyses. Consequently, the study population consisted of 1,220 respondents (403 Turkish, 326 Moroccan and 491 Dutch). Respondents with self-reported use of lipid lowering medication (n = 80) were excluded from analyses of mean cholesterol levels. To adjust for oversampling of migrant and older groups, population means and prevalence rates were weighted for age, using the SPSS Complex Samples procedure. For each stratum (20 different age-ethnic groups) a weight variable was derived from the proportion in the total population (based on census information on age and ethnicity from January 2004) and the proportion in the sample, whereby weight equals population size divided by sample size times post hoc correction.

Differences between means were tested using Student's *t*-test, differences between frequencies were tested using Pearson's Chi-square tests. Since the age distribution in the Amsterdam population is not the same for all ethnic groups, for instance migrant groups are still relatively young [10], weighting for age is not sufficient in completely eliminating a possible age effect. Therefore, to eliminate the explanatory effect of age differentials between ethnic groups in serum cholesterol levels (total, HDL and total/HDL ratio), beta-coefficients for ethnicity were calculated in multivariate linear regression analyses standardising for age. In the following steps several (other) demographic, lifestyle and endogenous factors were entered into the models. This enabled us to further analyse ethnic differences in cholesterol levels and determine whether ethnicity remained a significant factor in explaining differences in cholesterol levels, after adjustment for known determinants of cholesterol levels and cardiovascular disease risk. All analyses were performed using SPSS 15.0 (SPSS for Windows, Rel. 15.0.1. 2006. Chicago: SPSS Inc.).

## Results

The total response rate was 45% (Dutch: 46%, Turks: 50%, Moroccans: 39%). In total 1,329 Amsterdam residents aged 18 years and older, of Dutch, Turkish and Moroccan origin participated in the study, of which 1,220 (491 Dutch, 403 Turks, 326 Moroccans) were included in these analyses.

Table 1 describes the population characteristics by ethnic group. Turkish and Moroccan women were younger than their Dutch counterparts. Compared to the Dutch, educational level was lower in Turkish and Moroccan migrants. Mean BMI and prevalence of diabetes were higher in the Turkish and Moroccan ethnic populations. The physical activity level among Turkish migrants and Moroccan women was relatively low. In women, smoking was less common in migrant groups. Among men, the Turkish were more often smokers than the Dutch, and the Moroccan less often. In the Turkish and Moroccan ethnic groups alcohol abstinence was more common than in the Dutch population.

**Table 1:** Sample characteristics by sex and ethnic group

	**Dutch**	**Turkish**	**Moroccan**
	
**Men**	(*n *= 202)	(*n *= 189)	(*n *= 181)
	
Age (y ± sd)	51.2 ± 14.9	48.7 ± 12.6	52.6 ± 13.4
Height (cm ± sd)	178.8 ± 7.4	168.5 ± 6.3***	171.6 ± 6.7***
Weight (kg ± sd)	81.2 ± 13.7	78.8 ± 12.4	78.1 ± 12.8
Body Mass Index (kg/m^2 ^± sd)	25.4 ± 4.2	27.8 ± 4.4***	26.6 ± 3.8**
Diabetes (%)	5.1	11.1*	19.3***
Alcohol, total abstinence (%)	9.5	74.8***	93.6***
Smokers, current (%)	36.0	46.8*	24.5*
Physically active^† ^(%)	65.3	51.3**	61.9
Educational level (low, %)	12.1	56.9***	64.9***
	
**Women**	(*n *= 289)	(*n *= 214)	(*n *= 145)
	
Age (y ± sd)	51.7 ± 15.5	43.3 ± 14.0***	44.7 ± 13.9***
Height (cm ± sd)	165.9 ± 7.3	156.9 ± 5.6***	159.3 ± 6.5***
Weight (kg ± sd)	71.4 ± 13.4	74.2 ± 14.0*	75.1 ± 16.3*
Body Mass Index (kg/m^2 ^± sd)	26.0 ± 4.8	30.2 ± 5.8***	29.6 ± 6.3***
Diabetes (%)	3.2	8.0*	15.6***
Alcohol, total abstinence (%)	10.5	88.3***	93.2***
Smokers, current (%)	31.6	23.7	5.1***
Physically active^† ^(%)	71.6	37.4***	35.9***
Educational level (low, %)	22.0	65.6***	64.8***

sd, standard deviation

^†^at least 30 minutes of moderate physical activity on at least 5 days a week

*p < 0.05, ** *p *< 0.01, *** *p *< 0.001 (*t*-test for means, Chi-squared test for prevalence rates) significant difference compared with Dutch ethnic group

As shown in table 2, mean levels of total and HDL cholesterol were lower in the Turkish and Moroccan than in the Dutch ethnic population. Consequently, hypercholesterolemia was more prevalent among the Dutch and the prevalence of low HDL cholesterol was higher among the Turkish and Moroccans. Mean total/HDL cholesterol ratios were especially high in the Turkish population and also among Moroccan women. However, as compared to their Dutch counterparts a high total/HDL cholesterol ratio was significantly more prevalent among Turkish women, only.

**Table 2:** Mean cholesterol levels^$ ^and prevalence rates^$ ^of hypercholesterolemia and dyslipidemia by sex and ethnic group

	**Dutch**	**Turkish**	**Moroccan**
	
**Men**	(*n *= 202)	(*n *= 189)	(*n *= 181)
	
total cholesterol (mmol/l)	5.51 (5.33-5.70)	4.99 (4.78-5.19)***	4.71 (4.46-4.96)***
hypercholesterolemia^† ^(%)	24.1 (18.6-30.6)	8.9 (6.4-12.2)***	7.6 (4.8-11.8)***
HDL cholesterol (mmol/l)	1.40 (1.34-1.45)	1.14 (1.09-1.19)***	1.15 (1.09-1.22)***
low HDL cholesterol^‡ ^(%)	7.4 (4.0-13.3)	28.7 (19.9-39.4)***	25.6 (16.0-38.5)**
total/HDL cholesterol ratio	4.14 (3.94-4.34)	4.58 (4.34-4.82)**	4.29 (4.00-4.57)
high total/HDL cholesterol ratio^# ^(%)	21.2 (15.2-28.8)	30.0 (21.2-40.6)	28.3 (19.3-39.4)
	
**Women**	(*n *= 289)	(*n *= 214)	(*n *= 145)
	
total cholesterol (mmol/l)	5.38 (5.25-5.51)	5.01 (4.85-5.16)***	4.88 (4.71-5.05)***
hypercholesterolemia^† ^(%)	21.9 (18.7-25.5)	10.5 (6.9-15.6)***	8.3 (5.4-12.6)***
HDL cholesterol (mmol/l)	1.69 (1.63-1.75)	1.39 (1.33-1.45)***	1.40 (1.34-1.46)***
low HDL cholesterol^‡ ^(%)	11.1 (7.2-16.7)	39.4 (32.1-47.2)***	23.8 (16.6-32.9)**
total/HDL cholesterol ratio	3.37 (3.23-3.50)	3.84 (3.67-4.01)***	3.64 (3.46-3.82)*
high total/HDL cholesterol ratio^# ^(%)	7.9 (5.2-11.9)	19.3 (14.0-25.9)***	9.8 (5.9-15.8)

^$^weighted for age to adjust for oversampling, according to the age distribution in the Amsterdam population (with 95% confidence interval)

^†^Total serum cholesterol ≥6.5 mmol/l or use of lipid lowering medication

^‡ ^HDL cholesterol <1.0 mmol/l in men and <1.2 mmol/l in women

^#^Total/HDL cholesterol ratio >5

*p < 0.05, **p < 0.01, ***p < 0.001 (*t*-test for means, Chi-squared test for prevalence rates) significant difference compared with Dutch ethnic group

The data presented in table 2 were weighted for age according to the age distribution in Amsterdam with the aim of making each ethnic group in the sample by age similar to the ethnic group in the population. However, this does not correct for the fact that migrant groups in the population have a lower mean age compared to the non-migrant population. To eliminate the explanatory effect of age differentials between ethnic groups in serum cholesterol levels we performed multivariate regression analyses. To gain insight into other possible explanations for ethnic differences several demographic, endogenous and lifestyle factors were additionally introduced in the models as shown in tables 3, 4 and 5. Even after controlling for age, levels of total and HDL cholesterol remained relatively low, with significant negative beta-coefficients for ethnicity, among Turks and Moroccans and the resulting total/HDL cholesterol ratio relatively high (beta's for ethnicity remained significant and positive). In men, the other investigated factors had little effect on ethnic differences in total cholesterol. Ethnic differences in HDL cholesterol levels were substantially attenuated after controlling for ethnic differences in alcohol abstinence. Differences in the total/HDL cholesterol ratio among men with different ethnic backgrounds were attenuated after adjustment for BMI and alcohol abstinence, and in Turkish men adjusting for smoking had an additional attenuating effect. As a result ethnic differences in the total/HDL cholesterol ratio between Turkish and Moroccan men and Dutch men were no longer significant. In women, controlling for age, SES, smoking and alcohol abstinence substantially attenuated ethnic differences in total cholesterol levels; they were no longer significant. After adjusting for BMI and alcohol abstinence ethnic differences in HDL cholesterol levels in women attenuated. Controlling for BMI and alcohol abstinence also had an attenuating effect on the difference in total/HDL cholesterol ratio between Moroccan and Dutch women. In Turkish women, the investigated factors had little to no effect on ethnic differences, and only in this group the total/HDL cholesterol ratio remained significantly higher than in Dutch women.

**Table 3:** Multivariate linear regression analysis of the association between serum total cholesterol levels and ethnicity (B, 95%CI)

	**Dutch**	**Turkish**	**Moroccan**	**R**^**2**^
	
**Men**	(*n *= 172^†^)	(*n *= 164^†^)	(*n *= 162^†^)	
	
*Crude*	ref	-0.47 (-0.69 - -0.25)***	-0.56 (-0.78 - -0.34)***	0.06
*Model 1: adjusted for demographic factors*				
age	ref	-0.45 (-0.66 - -0.23)***	-0.61 (-0.82 - -0.39)***	0.10
age, socioeconomic status	ref	-0.45 (-0.70 - -0.20)***	-0.61 (-0.86 - -0.36)***	0.11
*Model 2: adjusted for demographic (age, SES) and endogenous factors*				
Body Mass Index	ref	-0.59 (-0.84 - -0.35)***	-0.65 (-0.89 - -0.40)***	0.15
Body Mass Index, diabetes	ref	-0.61 (-0.86 - -0.36)***	-0.63 (-0.89 - -0.38)***	0.16
*Model 3: adjusted for demographic (age, SES), endogenous (BMI, diabetes) and lifestyle factors*				
smoking	ref	-0.60 (-0.87 - -0.34)***	-0.60 (-0.87 - -0.34)***	0.16
smoking, alcohol abstinence	ref	-0.53 (-0.85 - -0.21)**	-0.50 (-0.85 - -0.15)**	0.15
smoking, alcohol abstinence, physical activity	ref	-0.50 (-0.82 - -0.17)**	-0.48 (-0.83 - -0.13)**	0.16
	
**Women**	(*n *= 243^†^)	(*n *= 193^†^)	(*n *= 134^†^)	
	
*Crude*	ref	-0.55 (-0.75 - -0.35)***	-0.60 (-0.83 - -0.38)***	0.07
*Model 1: adjusted for demographic factors*				
age	ref	-0.34 (-0.53 - -0.15)**	-0.42 (-0.63 - -0.21)***	0.22
age, socioeconomic status	ref	-0.27 (-0.50 - -0.05)*	-0.35 (-0.58 - -0.11)**	0.23
*Model 2: adjusted for demographic (age, SES) and endogenous factors*				
Body Mass Index	ref	-0.29 (-0.52 - -0.06)*	-0.34 (-0.58 - -0.10)**	0.25
Body Mass Index, diabetes	ref	-0.29 (-0.52 - -0.05)*	-0.34 (-0.59 - -0.09)**	0.26
*Model 3: adjusted for demographic (age, SES), endogenous (BMI, diabetes) and lifestyle factors*				
smoking	ref	-0.18 (-0.43 - 0.07)	-0.22 (-0.49 - 0.05)	0.28
smoking, alcohol abstinence	ref	-0.06 (-0.39 - 0.27)	-0.09 (-0.44 - 0.27)	0.26
smoking, alcohol abstinence, physical activity	ref	-0.07 (-0.40 - 0.27)	-0.09 (-0.44 - 0.27)	0.26

B, 95% CI, beta-coefficient with 95% confidence interval

R^2^, R-squared, explained variance of the model

^**†**^respondents with self-reported lipid lowering medication use excluded

* *p *< 0.05, ** *p *< 0.01, *** *p *< 0.001 significant difference compared with Dutch ethnic group

**Table 4:** Multivariate linear regression analysis of the association between serum HDL cholesterol levels and ethnicity (B, 95%CI)

	**Dutch**	**Turkish**	**Moroccan**	**R**^**2**^
	
**Men**	(*n *= 172^†^)	(*n *= 164^†^)	(*n *= 162^†^)	
	
*Crude*	ref	-0.25 (-0.33 - -0.18)***	-0.24 (-0.31 - -0.17)***	0.10
*Model 1: adjusted for demographic factors*				
age	ref	-0.26 (-0.33 - -0.19)***	-0.25 (-0.32 - -0.17)***	0.11
age, socioeconomic status	ref	-0.26 (-0.35 - -0.18)***	-0.23 (-0.31 - -0.14)***	0.13
*Model 2: adjusted for demographic (age, SES) and endogenous factors*				
Body Mass Index	ref	-0.23 (-0.31 - -0.14)***	-0.21 (-0.29 - -0.13)***	0.17
Body Mass Index, diabetes	ref	-0.23 (-0.32 - -0.15)***	-0.22 (-0.31 - -0.13)***	0.17
*Model 3: adjusted for demographic (age, SES), endogenous (BMI, diabetes) and lifestyle factors*				
smoking	ref	-0.21 (-0.30 - -0.12)***	-0.23 (-0.32 - -0.14)***	0.20
smoking, alcohol abstinence	ref	-0.12 (-0.23 - -0.01)*	-0.10 (-0.22 - 0.02)	0.24
smoking, alcohol abstinence, physical activity	ref	-0.12 (-0.23 - -0.01)*	-0.10 (-0.22 - 0.02)	0.24
	
**Women**	(*n *= 243^†^)	(*n *= 193^†^)	(*n *= 134^†^)	
	
*Crude*	ref	-0.35 (-0.43 - -0.28)***	-0.33 (-0.41 - -0.24)***	0.16
*Model 1: adjusted for demographic factors*				
age	ref	-0.37 (-0.45 - -0.29)***	-0.34 (-0.42 - -0.25)***	0.16
age, socioeconomic status	ref	-0.32 (-0.41 - -0.23)***	-0.29 (-0.39 - -0.20)***	0.17
*Model 2: adjusted for demographic (age, SES) and endogenous factors*				
Body Mass Index	ref	-0.23 (-0.32 - -0.14)***	-0.21 (-0.31 - -0.12)***	0.22
Body Mass Index, diabetes	ref	-0.22 (-0.31 - -0.13)***	-0.20 (-0.30 - -0.11)***	0.22
*Model 3: adjusted for demographic (age, SES), endogenous (BMI, diabetes) and factors*				
smoking	ref	-0.24 (-0.34 - -0.14)***	-0.23 (-0.34 - -0.12)***	0.23
smoking, alcohol abstinence	ref	-0.17 (-0.30 - -0.03)*	-0.12 (-0.26 - 0.03)	0.25
smoking, alcohol abstinence, physical activity	ref	-0.17 (-0.31 - -0.04)*	-0.12 (-0.26 - 0.02)	0.25

B, 95% CI, beta-coefficient with 95% confidence interval

^**†**^respondents with self-reported lipid lowering medication use excluded

* *p *< 0.05, ** *p *< 0.01, *** *p *< 0.001 significant difference compared with Dutch ethnic group

**Table 5:** Multivariate linear regression analysis of the association between total/HDL cholesterol ratio and ethnicity (B, 95%CI)

	**Dutch**	**Turkish**	**Moroccan**	**R**^**2**^
	
**Men**	(*n *= 172^†^)	(*n *= 164^†^)	(*n *= 162^†^)	
	
*Crude*	ref	0.60 (0.31 - 0.89)***	0.37 (0.08 - 0.66)*	0.03
*Model 1: adjusted for demographic factors*				
age	ref	0.64 (0.34 - 0.93)***	0.35 (0.06 - 0.64)*	0.05
age, socioeconomic status	ref	0.65 (0.32 - 0.98)***	0.30 (-0.04 - 0.63)	0.06
*Model 2: adjusted for demographic (age, SES) and endogenous factors*				
Body Mass Index	ref	0.42 (0.10 - 0.75)*	0.22 (-0.11 - 0.55)	0.13
Body Mass Index, diabetes	ref	0.44 (0.11 - -0.78)*	0.28 (-0.06 - -0.62)	0.14
*Model 3: adjusted for demographic (age, SES), endogenous (BMI, diabetes) and lifestyle factors*				
smoking	ref	0.35 (0.01 - 0.69)*	0.33 (-0.02 - 0.67)	0.17
smoking, alcohol abstinence	ref	0.18 (-0.25 - 0.61)	0.07 (-0.39 - 0.54)	0.19
smoking, alcohol abstinence, physical activity	ref	0.20 (-0.23 - 0.63)	0.09 (-0.38 - 0.55)	0.19
	
**Women**	(*n *= 243^†^)	(*n *= 193^†^)	(*n *= 134^†^)	
	
*Crude*	ref	0.52 (0.30 - 0.73)***	0.30 (0.06 - 0.54)*	0.04
*Model 1: adjusted for demographic factors*				
age	ref	0.71 (0.50 - 0.92)***	0.46 (0.23 - 0.69)***	0.14
age, socioeconomic status	ref	0.62 (0.38 - 0.86)***	0.39 (0.13 - 0.65)**	0.14
*Model 2: adjusted for demographic (age, SES) and endogenous factors*				
Body Mass Index	ref	0.40 (0.15 - 0.65)**	0.21 (-0.05 - 0.47)	0.20
Body Mass Index, diabetes	ref	0.38 (0.13 - 0.63)**	0.18 (-0.09 - 0.45)	0.20
*Model 3: adjusted for demographic (age, SES), endogenous (BMI, diabetes) and lifestyle factors*				
smoking	ref	0.52 (0.25 - 0.79)***	0.32 (0.03 - 0.62)*	0.22
smoking, alcohol abstinence	ref	0.46 (0.10 - 0.83)*	0.20 (-0.19 - 0.58)	0.22
smoking, alcohol abstinence, physical activity	ref	0.47 (0.10 - 0.84)*	0.20 (-0.19 - 0.59)	0.22

B, 95% CI, beta-coefficient with 95% confidence interval

^**†**^respondents with self-reported lipid lowering medication use excluded

* *p *< 0.05, ** *p *< 0.01, *** *p *< 0.001 significant difference compared with Dutch ethnic group

## Discussion

We found that total cholesterol levels are relatively low among the Moroccan and Turkish migrant populations and that hypercholesterolemia is less prevalent compared to the ethnic Dutch population. On the other hand levels of HDL cholesterol are also relatively low in these migrant groups. The resulting total/HDL cholesterol ratio is especially unfavourable among the Turkish ethnic group.

Literature on cardiovascular disease risk among Turkish and Moroccan migrants in Europe is scarce. Only a few population-based studies focusing on cholesterol levels among Turkish migrants have been conducted. One Dutch study conducted in the early nineties among a smaller sample of 149 Turkish migrants also reported relatively low total and HDL cholesterol levels, comparable to our findings [18]. Glenday et al. found, that Turkish migrants in Oslo, Norway had relatively low total and HDL cholesterol levels. Although they found slightly higher mean total cholesterol levels among Turkish men (5.50 mmol/l), they found similar levels of total cholesterol among Turkish women (5.16 mmol/l) and comparable mean HDL cholesterol levels among both men (1.14 mmol/l) and women (1.35 mmol/l) [19]. Studies conducted in Turkey have also shown relatively low levels of both total and HDL cholesterol among Turkish adults [20,21]. Tazi and colleagues reported relatively low levels of total cholesterol among Moroccan adults, living in Morocco [22]. To our knowledge, no (recent) population-based studies on cardiovascular disease risk among Moroccan migrants in Europe have been reported.

We investigated several demographic, endogenous and lifestyle factors as possible explanations for ethnic differences in cholesterol levels. Several of these factors indeed attenuate ethnic differences in cholesterol levels. However, these factors cannot explain ethnic differences in total cholesterol levels in men. In women, differences in age, SES and alcohol abstinence can almost completely be held accountable for the relatively low total cholesterol levels in Turkish and Moroccan women. In explaining ethnic differences in HDL cholesterol and the total/HDL cholesterol ratio, high levels of total alcohol abstinence in the Turkish and Moroccan ethnic groups and their relatively high BMI seem to be important factors. The high prevalence of smoking in Turkish men also appears to be relevant in explaining their high mean total/HDL cholesterol ratio. However, these factors cannot completely explain the relatively low HDL cholesterol levels among Turkish migrants and high total/HDL cholesterol ratio among Turkish women; differences with the Dutch group remained significant. Glenday et al. also found that ethnic differences in mean HDL cholesterol among Turkish migrants were attenuated but persisted after adjustment for BMI and waist-to-hip ratio [19].

### Strengths and limitations

Only limited information is available on cholesterol levels and dyslipidemia among Turkish and, especially, Moroccan migrant groups in western European countries such as the Netherlands. Although these groups are among the largest ethnic minority groups in several European countries they are often poorly represented in epidemiological studies. Our study presents information on total and HDL cholesterol levels from a population-based sample of Turkish and Moroccan migrants, including the older population. Additionally, our results give insight into possible explanations for ethnic differences in cholesterol levels.

The overall response rate is not very high (45%). However, it is comparable to that of several other large national surveys in the Netherlands, indicating that any systematic bias is unlikely [7,23,24]. Furthermore, the number of individuals who didn't receive the survey invitation is likely to be high, due to the high mobility of the Amsterdam population and incorrect residential information in the municipal population register (between 7.5% and 15% according to recent investigation [25]). This may have had a negative effect on our response rate. To correct for oversampling and non-response bias, population means and prevalence rates were weighted for age. Altogether, we consider these measures to be sufficient in reducing the effect of a possible selection bias.

Since fasting measurements are not feasible in a general health survey, blood samples were collected in a non fasting state. However food consumption within a few hours before blood collection does not have an immediate effect on serum total and HDL cholesterol levels [26].

## Conclusions

Total cholesterol levels are relatively low in Turkish and Moroccan migrant groups in the Netherlands. However part of this advantage is off-set by the fact that HDL cholesterol levels are also relatively low in these ethnic groups. The total/HDL cholesterol ratio is particularly unfavourable in the Turkish ethnic group. Important factors in explaining these ethnic differences are the fact that BMI, prevalence of alcohol abstinence, and, in Turkish men, smoking are all relatively high in these migrant groups. Interventions targeted on reducing overweight and smoking, are not only important in light of efforts to reduce overall cardiovascular disease risk, but also, more specifically in elevating HDL cholesterol levels and improving the total/HDL cholesterol ratio among Turkish and Moroccan migrants. Especially among Turks and Moroccans, who are at risk for CVD because of their low HDL cholesterol levels, screening should not merely be aimed at total cholesterol but also at HDL cholesterol and the total/HDL cholesterol ratio [27].

More research is necessary to gain insight into the impact of ethnic differences in HDL and total cholesterol on CVD morbidity and mortality. In addition, absolute heart disease risk at the same serum cholesterol levels is shown to be different across cultures. This indicates that although cholesterol is an important determinant there are other important risk factors [5]. Further study on the cardiovascular risk pattern of Turkish and Moroccan migrants should improve insight into cardiovascular risk and prevention of CVD mortality.

## Competing interests

The authors declare that they have no competing interests.

## Authors' contributions

JU participated in study concept, design and coordination, was responsible for statistical analyses and interpretation of data and drafted the manuscript. GB participated in analyses and interpretation of data, helped to draft the manuscript and was involved in its critical revision. CB participated in study concept and design and was involved in critical revision of the manuscript. DU participated in study concept, design and coordination, participated in analyses and interpretation of data and was involved in critical revision of the manuscript. AV participated in study concept and design and was involved in critical revision of the manuscript. JS participated in analyses and interpretation of data and was involved in critical revision of the manuscript. All authors read and approved the final manuscript.

## Pre-publication history

The pre-publication history for this paper can be accessed here:

http://www.biomedcentral.com/1471-2458/10/740/prepub

## Supplementary Material

Additional file 1 **Research questionnaire**. English translation of the questionnaire used during the health interview. Click here for file
