# Supplementary material for: Ethnic differences in total and HDL cholesterol among Turkish, Moroccan and Dutch ethnic groups living in Amsterdam, the Netherlands
Source: BMC Public Health. 2010 Nov 30;10:740. doi: 10.1186/1471-2458-10-740 (PMC3002334; doi:10.1186/1471-2458-10-740)
Supplement: Additional file 1 — Research questionnaire. English translation of the questionnaire used during the health interview. [file 1471-2458-10-740-S1.DOC]

###### PART TO BE FILLED IN BY THE INTERVIEWER

N.B. Fill in the respondent number on each sheet of this questionnaire of the respondent sitting in front of you. Use the blocks at the top right of each sheet.

INT. Below, fill in the date on which the interview is held

| date |  |  | - |  |  | - | *2* | *0* | *0* | *4* |
| --- | --- | --- | --- | --- | --- | --- | --- | --- | --- | --- |

INT. Fill in the time when you start this interview

| time |  |  | : |  |  |
| --- | --- | --- | --- | --- | --- |

INT. Fill in the interviewer number below

| Interviewer number |  |  |  |  |  |
| --- | --- | --- | --- | --- | --- |

INT. Fill in the number of the clinic below

| Location number |  |  |
| --- | --- | --- |

Now start the interview!

###### GENERAL

tekst-alg1: First of all, I would like to ask you some general questions about your family situation.

alg1 – Respondent’s gender [INT: fill in yourself]

1. ❑ male
2. ❑ female

alg2 – In what year were you born?

| year: |  |  |  |  | 97. ❑ don’t know |
| --- | --- | --- | --- | --- | --- |

alg3a - What is your country of birth? INT. WRITE ANSWER IN COLUMN A OF TABLE 1

alg3b - What is your mother’s country of birth? INT. WRITE ANSWER IN COLUMN B OF TABLE 1

alg3c - What is your father’s country of birth? INT. WRITE ANSWER IN COLUMN C OF TABLE 1

TABLE 1 Country of birth

|  | A | B | C |
| --- | --- | --- | --- |
|  | country of birth respondent | country of birth mother | country of birth father |
| 1. Netherlands | 1. ❑ | 1. ❑ | 1. ❑ |
| 1. Surinam | 2. ❑ | 2. ❑ | 2. ❑ |
| 1. Netherlands Antilles | 3. ❑ | 3. ❑ | 3. ❑ |
| 1. Aruba | 4. ❑ | 4. ❑ | 4. ❑ |
| 1. Turkey | 5. ❑ | 5. ❑ | 5. ❑ |
| 1. Morocco | 6. ❑ | 6. ❑ | 6. ❑ |
| 1. other, namely | 7. ❑  ……………………………. | 7. ❑  ……………………………. | 7. ❑  ……………………………. |
| 1. don’t know | 97. ❑ | 97. ❑ | 97. ❑ |
| 1. no answer | 98. ❑ | 98. ❑ | 98. ❑ |
| 1. not applicable | 99. ❑ | 99. ❑ | 99. ❑ |

[INT. SHOW CARD 1]

alg4 – What is your population group? [INT: only one answer possible]

1. ❑ Dutch
2. ❑ Surinamese
3. ❑ Creole Surinamese
4. ❑ Hindustani Surinamese
5. ❑ Chinese Surinamese
6. ❑ Javanese Surinamese
7. ❑ Antillean
8. ❑ Aruban
9. ❑ Moroccan
10. ❑ Berber
11. ❑ Turkish
12. ❑ Kurdish
13. ❑ other, namely…………………………………………………………………………………………………………………

97. ❑ d.k. 98. ❑ n.a. 99. ❑ N/A

*Int. Only ask alg5 if the respondent was not born in the Netherlands.*

alg5 – How long have you been living in the Netherlands?

| year: |  |  |  |  | 97. ❑ don’t know |
| --- | --- | --- | --- | --- | --- |

[INT.: SHOW CARD 2]

alg6 **–** When you look at this card, can you indicate which situation currently applies to you?

[INT.: more than one answer possible. INT.: Children or not comes up in a later question - alg10!]

1. ❑ I live with a partner
2. ❑ I have a lasting relationship, but do not live with a partner
3. ❑ I live alone
4. ❑ I live with my parent(s)/guardian(s)
5. ❑ a different situation applies to me

97. ❑ d.k. 98. ❑ n.a. 99. ❑ N/A

*INT. Only ask the following questions -alg7a-c - if the respondent lives together or has a lasting relationship (alg6 = 1 of 2)*

alg7a **–** What is the gender of your partner?

1. ❑ male
2. ❑ female

97. ❑ d.k. 98. ❑ n.a. 99. ❑ N/A

[INT. SHOW CARD 1]

alg7b – Can you indicate the population group of your partner on the basis of this card?

1. ❑ Dutch
2. ❑ Surinamese
3. ❑ Creole Surinamese
4. ❑ Hindustani Surinamese
5. ❑ Chinese Surinamese
6. ❑ Javanese Surinamese
7. ❑ Antillean
8. ❑ Aruban
9. ❑ Moroccan
10. ❑ Berber
11. ❑ Turkish
12. ❑ Kurdish
13. ❑ other, namely………………………………………………………………………………………………………………...

97. ❑ d.k. 98. ❑ n.a. 99. ❑ N/A

alg7c - Is your partner your cousin, your uncle *or* aunt, or your half-brother *or* half-sister?

1. ❑ yes, cousin
2. ❑ yes, uncle/aunt
3. ❑ yes, half-brother/half-sister
4. ❑ no

97. ❑ d.k. 98. ❑ n.a. 99. ❑ N/A

*INT. The next questions (alg8-alg11b) are only to be asked if the respondent does not live alone (alg6=3).*

*If the respondent lives alone, go to the next block: tekst-sf1*

alg8 – How many persons make up your household (including yourself)?

|  |  | persons | 98. ❑ no answer |
| --- | --- | --- | --- |

INT. HOME = 77

alg9 – How many of them are 16 or older (including yourself)?

|  |  | persons | 98. ❑ no answer |
| --- | --- | --- | --- |

alg10 – Can you tell me how this household is composed?

1. ❑ two adults, no children
2. ❑ (married) couple with child(ren) living at home
3. ❑ SINGLE parent with child(ren) living at home
4. ❑ other, with child(ren) living at home
5. ❑ other, without child(ren) living at home

97. ❑ d.k. 98. ❑ n.a. 99. ❑ N/A

*INT. The next questions (alg11a-b) are only to be asked if children living at home are present in the household (alg10=2/3/4)*

alg11a – How many children living at home are there in this household?

|  |  | children | 98. ❑ no answer |
| --- | --- | --- | --- |

alg11b – How old are the children living at home?

INT. WRITE THE AGES IN YEARS IN THE DIAGRAM BELOW INT. START WITH THE OLDEST CHILD: How old is your oldest child living at home?, THEN THE SECOND, THIRD CHILD ETC. ETC.

|  | age in years | | | |  | | | | | | | | | | | | | | | | | |
| --- | --- | --- | --- | --- | --- | --- | --- | --- | --- | --- | --- | --- | --- | --- | --- | --- | --- | --- | --- | --- | --- | --- |
|  | |  | |  | | | | | | | | | | | | | | | | | | |
| 1. ❑ oldest child | |  |  |  | | age in years | | | |  | | | | | | | | | | | | |
|  | |  |  |  | | |  |  |  | | | | | | | | | | | | | |
| 2. ❑ 2^nd^ child | |  |  | 4. ❑ 4^th^ child | | |  |  |  | | age in years | | | | | |  | | | | | |
|  | |  |  |  | | |  |  |  | | |  | |  | |  | | | | | | |
| 3. ❑ 3^rd^ child | |  |  | 5. ❑ 5^th^ child | | |  |  | 7. ❑ 7^th^ child | | |  |  | |  | | | age in years | | | | |
|  | |  |  |  | | |  |  |  | | |  |  | |  | | | |  |  | |  |
|  | |  |  | 6. ❑ 6^th^ child | | |  |  | 8. ❑ 8^th^ child | | |  |  | | 9. ❑ 9^th^ child | | | |  |  |  | |
|  | |  |  |  | | |  |  |  | | |  |  | |  | | | |  |  |  | |
| 97. ❑ d.k. 98. ❑ n.a. 99. ❑ N/A | | | | | | | | | | | | | | | 10. ❑ 10^th^ child | | | |  |  |  | |

**HEALTH AS EXPERIENCED**

tekst-sf1: I am now going to ask you some questions about your health and things having to do with it.

[INT. SHOW CARD 3]

sf01 – In general, would you say your health is? Look at this card for the possible answers.

1. ❑ excellent
2. ❑ very good
3. ❑ good
4. ❑ fair
5. ❑ poor

97. ❑ d.k. 98. ❑ n.a. 99. ❑ N/A

tekst-sf2: The following items are about activities you might do during a typical day. Does your health now limit you in these activities? If so, how much?

sf02 - Moderate activities, such as moving a table, pushing a vacuum cleaner, bowling or playing golf

1. ❑ Yes, Limited A Lot
2. ❑ Yes, Limited A Little
3. ❑ No, not limited at all
4. ❑ Does not engage in these activities

97. ❑ d.k. 98. ❑ n.a. 99. ❑ N/A

sf03 – Climbing several flights of stairs.

1. ❑ Yes, Limited A Lot
2. ❑ Yes, Limited A Little
3. ❑ No, not limited at all
4. ❑ Does not engage in these activities

97. ❑ d.k. 98. ❑ n.a. 99. ❑ N/A

tekst-sf3: During the **past 4 weeks,** have you had any of the following problems with your work or other regular daily activities as a result of your physical health

sf04 - **Accomplished less** than you would like?

1. ❑ yes
2. ❑ no

97. ❑ d.k. 98. ❑ n.a. 99. ❑ N/A

sf05 – Were limited in the **kind** of work or other activities?

1. ❑ yes
2. ❑ no

97. ❑ d.k. 98. ❑ n.a. 99. ❑ N/A

tekst-sf4: During the **past 4 weeks,** have you had any of the following problems with your work or other regular daily activities as a result of enotional problems (such as feeling depressed or anxious)?

sf06 - **Accomplished less** than you would like?

1. ❑ yes
2. ❑ no

97. ❑ d.k. 98. ❑ n.a. 99. ❑ N/A

sf07 – Didn't do your work or other acticvities as **carefully** as usual

1. ❑ yes
2. ❑ no

97. ❑ d.k. 98. ❑ n.a. 99. ❑ N/A

[INT. SHOW CARD 4]

sf08 –During the past four weeks, how much did pain interfere with your normal work (including both work outside the home and housework)?

1. ❑ Not at all
2. ❑ A little bit
3. ❑ Moderately
4. ❑ Quite a bit
5. ❑ Extremely

97. ❑ d. k. 98. ❑ n.a. 99. ❑ N/A

tekst-sf5: These questions are about how you feel and how things have been with you during the **past 4 weeks**. For each question, please give the answer closests to the way you have been feeling. How much of the time during the **past 4 weeks**

[INT. SHOW CARD 5 AND WRITE THE ANSWERS TO THE FOLLOWING QUESTIONS IN TABLE 7]

sf09 – Have you been a very nervous person?

sf10 – How you felt so down in the dumps nothing could cheer you up?

sf11 – Have you felt calm and peaceful?

sf12 – Did you have a lot of energy?

sf13 – Have you felt downhearted and blue?

sf14 – Have you been a happy person?

sf15 - Has your physical health or emotional problems interfered with your social activities (like visiting with friends, relatives etc.)?

| TABLE 2 | All of the time | Most of the time | A good bit of the time | Some of the time | A little of the time | None of the time | 97. 98. 99. |
| --- | --- | --- | --- | --- | --- | --- | --- |
| sf09…very nervous | 1. ❑ | 2. ❑ | 3. ❑ | 4. ❑ | 5. ❑ | 6. ❑ | ❑ d.k. ❑ n.a. ❑ N/A |
| sf10…down in the dumps | 1. ❑ | 2. ❑ | 3. ❑ | 4. ❑ | 5. ❑ | 6. ❑ | ❑ d.k. ❑ n.a. ❑ N/A |
| sf11…felt calm and peaceful? | 1. ❑ | 2. ❑ | 3. ❑ | 4. ❑ | 5. ❑ | 6. ❑ | ❑ d.k. ❑ n.a. ❑ N/A |
| sf12…Did you have a lot of energy? | 1. ❑ | 2. ❑ | 3. ❑ | 4. ❑ | 5. ❑ | 6. ❑ | ❑ d.k. ❑ n.a. ❑ N/A |
| sf13…felt downhearted and blue? | 1. ❑ | 2. ❑ | 3. ❑ | 4. ❑ | 5. ❑ | 6. ❑ | ❑ d.k. ❑ n.a. ❑ N/A |
| sf14…happy person | 1. ❑ | 2. ❑ | 3. ❑ | 4. ❑ | 5. ❑ | 6. ❑ | ❑ d.k. ❑ n.a. ❑ N/A |
| sf15…interfered with social activities | 1. ❑ | 2. ❑ | 3. ❑ | 4. ❑ | 5. ❑ | 6. ❑ | ❑ d.k. ❑ n.a. ❑ N/A |

###### CONTACT WITH MEDICAL HEALTHCARE PROVIDERS

tekst-med1: I would now like to ask you some questions about contact with healthcare providers.

med1a – Have you contacted a general practitioner in the last 2 months about yourself?
[INT.: INCLUDE contact WITH SUBSTITUTES AND BY TELEPHONE. ]

1. ❑ yes 🡪 go to **med1b**
2. ❑ no 🡪 go to **med2**

97. ❑ d.k. 98. ❑ n.a. 99. ❑ N/A

med1b – How often have you contacted a general practitioner in the last 2 months about yourself?
[INT.: If necessary, estimate the number of times]

|  |  | time(s) | 97. ❑ d.k. 98. ❑ n.a. 99. ❑ N/A |
| --- | --- | --- | --- |

[INT. SHOW CARD 4]

med1c – What are or were the reasons for the most recent contact with the general practitioner? Please look at this card.

[INT.: mORE THAN ONE ANSWER POSSIBLE]

1. ❑ a complaint, namely.………………………………………………………………………………………………………..
2. ❑ information and advice
3. ❑ refill of a prescription
4. ❑ check-up
5. ❑ contraceptives / anti-conception means
6. ❑ to have a pap smear made
7. ❑ vaccination
8. ❑ birth/ pregnancy check-up
9. ❑ other, namely ………………………………………………………………………………………………………………..

97. ❑ d.k. 98. ❑ n.a. 99. ❑ N/A

med2 – Have you contacted a medical specialist in the last 2 months about yourself on an outpatient basis?
[INT.: do not include admissions]

1. ❑ yes
2. ❑ no

97. ❑ d.k. 98. ❑ n.a. 99. ❑ N/A

med3 – Have you been in a hospital or clinic at some time in the last **12** months?

1. ❑ yes
2. ❑ no

97. ❑ d.k. 98. ❑ n.a. 99. ❑ N/A

med4 – Have you been treated for psychological problems in the last **12** months?

1. ❑ yes 🡪 go to **med5**
2. ❑ no 🡪 go to **medic1**

97. ❑ d.k. 98. ❑ n.a. 99. ❑ N/A

med5 – Where were you treated for psychological problems? [INT.: more than one answer possible, do not read aloud!]

1 ❑ Family doctor

2 ❑ Company doctor

3 ❑ Regional institute for mental welfare (RIAGG)

4 ❑ Emergency centre

5 ❑ Psychiatric hospital

6 ❑ General social services

7 ❑ Psychiatric outpatients' clinic of the hospital

8 ❑ Consultation service for alcohol and drugs (CAD)

9 ❑ Psychiatrist's, psychologist's or psychotherapist's practice

10 ❑ Alternative treatment

11 ❑ Traditional healer

12 ❑ Physiotherapist, haptonomist

13 ❑ elsewhere, namely:

97. ❑ d.k. 98. ❑ n.a. 99. ❑ N/A

medic1 – Are you using medicines or vitamin and/or mineral supplements at present?

1. ❑ yes 🡪 go to **medic2**
2. ❑ no 🡪 go to the next block, **text11**

97. ❑ d.k. 98. ❑ n.a. 99. ❑ N/A

medic2 – Which products are these?

1. ❑ Product 1…………………………………………………………………………………………………………………………
2. ❑ Product 2…………………………………………………………………………………………………………………………
3. ❑ Product 3…………………………………………………………………………………………………………………………
4. ❑ Product 4…………………………………………………………………………………………………………………………

97. ❑ d.k. 98. ❑ n.a. 99. ❑ N/A

###### CHRONIC DISORDERS

Text11 – The next questions are about chronic diseases.

chron1a – Have you ever had a **stroke, brain haemorrhage or cerebral infarction**?

1. ❑ yes 🡪 go to **chron1b**
2. ❑ no 🡪 go to **chron2a**

97. ❑ d.k. 98. ❑ n.a. 99. ❑ N/A

chron1b – Did you have this in the past 12 months?

1. ❑ yes
2. ❑ no

97. ❑ d.k. 98. ❑ n.a. 99. ❑ N/A

chron1c – Have you been undergoing treatment or medical supervision for this by your family family doctor or a specialist in the past 12 months?

1. ❑ yes
2. ❑ no

97. ❑ d.k. 98. ❑ n.a. 99. ❑ N/A

chron1d – Are you still experiencing health problems or limitations as a result of that?

1. ❑ yes
2. ❑ no

97. ❑ d.k. 98. ❑ n.a. 99. ❑ N/A

chron2a – Have you ever had a **heart attack**?

1. ❑ yes 🡪 go to **chron2b**
2. ❑ no 🡪 go to **chron3a**

97. ❑ d.k. 98. ❑ n.a. 99. ❑ N/A

chron2b – Did you have this in the past 12 months?

1. ❑ yes
2. ❑ no

97. ❑ d.k. 98. ❑ n.a. 99. ❑ N/A

chron2c – Have you been undergoing treatment or medical supervision for this by your family family doctor or a specialist in the past 12 months?

1. ❑ yes
2. ❑ no

97. ❑ d.k. 98. ❑ n.a. 99. ❑ N/A

chron2d – Are you still experiencing health problems or limitations as a result of that?

1. ❑ yes
2. ❑ no

97. ❑ d.k. 98. ❑ n.a. 99. ❑ N/A

chron3a – Have you ever had **another serious heart disorder (such as heart failure or angina pectoris)**?

1. ❑ yes 🡪 go to **chron3b**
2. ❑ no 🡪 go to **chron4a**

97. ❑ d.k. 98. ❑ n.a. 99. ❑ N/A

chron3b – Did you have this in the past 12 months?

1. ❑ yes
2. ❑ no

97. ❑ d.k. 98. ❑ n.a. 99. ❑ N/A

chron3c – Have you been undergoing treatment or medical supervision for this by your family family doctor or a specialist in the past 12 months?

1. ❑ yes
2. ❑ no

97. ❑ d.k. 98. ❑ n.a. 99. ❑ N/A

chron3d – Are you still experiencing health problems or limitations as a result of that?

1. ❑ yes
2. ❑ no

97. ❑ d.k. 98. ❑ n.a. 99. ❑ N/A

chron4a – Have you ever had any form of cancer (malignant disorder)?

1. ❑ yes 🡪 go to **chron4b**
2. ❑ no 🡪 go to **chron5a**

97. ❑ d.k. 98. ❑ n.a. 99. ❑ N/A

chron4b – Did you have this in the past 12 months?

1. ❑ yes
2. ❑ no

97. ❑ d.k. 98. ❑ n.a. 99. ❑ N/A

chron4c – Have you been undergoing treatment or medical supervision for this by your family family doctor or a specialist in the past 12 months?

1. ❑ yes
2. ❑ no

97. ❑ d.k. 98. ❑ n.a. 99. ❑ N/A

chron4d – Are you still experiencing health problems or limitations as a result of this?

1. ❑ yes
2. ❑ no

97. ❑ d.k. 98. ❑ n.a. 99. ❑ N/A

chron5a – Do you have diabetes?

1. ❑ yes 🡪 go to **chron5b**
2. ❑ no 🡪 go to **chron6a**

97. ❑ d.k. 98. ❑ n.a. 99. ❑ N/A

chron5b – Have you been undergoing treatment or medical supervision for this by your family family doctor or a specialist in the past 12 months? 

1. ❑ yes
2. ❑ no

97. ❑ d.k. 98. ❑ n.a. 99. ❑ N/A

chron5c – Are you using insulin or tablets for this at present?

1. ❑ yes, insulin 🡪 go to **chron5d**
2. ❑ yes, tablets 🡪 go to **chron5e**
3. ❑ no 🡪 go to **chron5e**

97. ❑ d.k. 98. ❑ n.a. 99. ❑ N/A

chron5d – Did you start taking insulin within 6 months of your having been diagnosed with diabetes?

1. ❑ yes
2. ❑ no

97. ❑ d.k. 98. ❑ n.a. 99. ❑ N/A

chron5e – Are you now on a special diet for this?

1. ❑ yes
2. ❑ no

97. ❑ d.k. 98. ❑ n.a. 99. ❑ N/A

#### chron6a – Do you now have, or have you had high blood pressure in the last 12 months?

1. ❑ yes 🡪 NOTE: go to **chron6b**
2. ❑ no 🡪 NOTE: go to **chron6b**

97. ❑ d.k. 98. ❑ n.a. 99. ❑ N/A

#### chron6b – Have you been undergoing treatment or medical supervision for this by your family family doctor or a specialist in the past 12 months?

1. ❑ yes
2. ❑ no

97. ❑ d.k. 98. ❑ n.a. 99. ❑ N/A

#### chron6c –Do you use medicines to lower your blood pressure?

1. ❑ yes
2. ❑ no

97. ❑ d.k. 98. ❑ n.a. 99. ❑ N/A

#### chron6d – Are you now on a diet to lower your blood pressure?

1. ❑ yes
2. ❑ no

97. ❑ d.k. 98. ❑ n.a. 99. ❑ N/A

#### chron7a – Do you now have, or have you had high cholesterol in the last 12 months?

1. ❑ yes 🡪 NOTE: go to **chron7b**
2. ❑ no 🡪 NOTE: go to **chron7b**

97. ❑ d.k. 98. ❑ n.a. 99. ❑ N/A

#### chron7b – Have you been undergoing treatment or medical supervision for this by your family family doctor or a specialist in the past 12 months?

1. ❑ yes
2. ❑ no

97. ❑ d.k. 98. ❑ n.a. 99. ❑ N/A

#### chron7c – Are you now taking medicines to lower your cholesterol?

1. ❑ yes
2. ❑ no

97. ❑ d.k. 98. ❑ n.a. 99. ❑ N/A

#### chron7d – Are you now on a diet to lower your cholesterol?

1. ❑ yes
2. ❑ no

97. ❑ d.k. 98. ❑ n.a. 99. ❑ N/A

chron8 – Could you indicate if you had the following diseases and disorders in **the past 12 months**? Please also indicate whether or not this disease or disorder was diagnosed by a doctor.

|  | no | | yes, **not** diagnosed by a doctor | | Yes, diagnosed by a doctor | | 97. 98. 99. |
| --- | --- | --- | --- | --- | --- | --- | --- |
| 1. migraine or frequent serious headache | 1. ❑ | | 2. ❑ | | 3. ❑ | | ❑ d.k. ❑ n.a. ❑ N/A |
| 1. stricture of the blood vessels in the stomach or legs (**no** varicose veins) | 1. ❑ | | 2. ❑ | | 3. ❑ | | ❑ d.k. ❑ n.a. ❑ N/A |
| 1. asthma, chronic bronchitis, pulmonary emphysema or CNSLD (Chronic Non-Specific Lung Disease) | 1. ❑ | | 2. ❑ | | 3. ❑ | | ❑ d.k. ❑ n.a. ❑ N/A |
| 1. serious or obstinate intestinal disorders lasting more than 3 months | 1. ❑ | | 2. ❑ | | 3. ❑ | | ❑ d.k. ❑ n.a. ❑ N/A |
| 1. psoriasis | 1. ❑ | | 2. ❑ | | 3. ❑ | | ❑ d.k. ❑ n.a. ❑ N/A |
| 1. chronic eczema | | 1. ❑ | | 2. ❑ | | 3. ❑ | ❑ d.k. ❑ n.a. ❑ N/A |
| 1. involuntary loss of urine (incontinence) | | 1. ❑ | | 2. ❑ | | 3. ❑ | ❑ d.k. ❑ n.a. ❑ N/A |
| 1. serious or persistent back complaints (including slipped disc) | | 1. ❑ | | 2. ❑ | | 3. ❑ | ❑ d.k. ❑ n.a. ❑ N/A |
| 1. articular degeneration (arthrosis, erosive rheumatism) of hips and knees | | 1. ❑ | | 2. ❑ | | 3. ❑ | ❑ d.k. ❑ n.a. ❑ N/A |
| 1. chronic inflammation of the joints (inflammatory arthritis, chronic rheumatism, rheumatoid arthritis) | | 1. ❑ | | 2. ❑ | | 3. ❑ | ❑ d.k. ❑ n.a. ❑ N/A |
| 1. other serious or obstinate neck or shoulder disorder | | 1. ❑ | | 2. ❑ | | 3. ❑ | ❑ d.k. ❑ n.a. ❑ N/A |
| 1. other serious elbow, wrist, or hand disorder | | 1. ❑ | | 2. ❑ | | 3. ❑ | ❑ d.k. ❑ n.a. ❑ N/A |
| 1. other long illness or disorder, namely: ………………………………………………………… | | 1. ❑ | | 2. ❑ | | 3. ❑ | ❑ d.k. ❑ n.a. ❑ N/A |

soa1 – Have you ever had the venereal disease(s) Gonorrhoea, Syphilis, Chlamydia or Herpes?

1. ❑ No, never 🡪 go to the next block, **tekst-ongev**
2. ❑ Yes, in the past year 🡪 go to **soa2**
3. ❑ Yes, more than 1 year ago 🡪 go to the next block, **tekst-ongev**

97. ❑ d.k. 98. ❑ n.a. 99. ❑ N/A

soa2 – Did you get treatment for this?

1. ❑ yes
2. ❑ no

97. ❑ d.k. 98. ❑ n.a. 99. ❑ N/A

###### ACCIDENTS

### text-ongev: The next questions are about accidents.

### ongev1 – Have you suffered bodily harm, poisoning or an injury one or more times in the last 12 months?

### This means bodily harm, poisoning and injuries that occurred suddenly and received medical treatment; in other words for which you went to a hospital, first-aid station, doctor or physical therapist.

1. ❑ yes, once 🡪 go to **ongev2**
2. ❑ yes, twice 🡪 go to **ongev2**
3. ❑ yes, more than twice 🡪 go to **ongev2**
4. ❑ no 🡪 go to the next block, **adl1**

97. ❑ d.k. 98. ❑ n.a. 99. ❑ N/A

[INT. SHOW CARD 7]

ongev2 – This bodily harm or injury occurred [INT. SHOW CARD 7, mORE THAN ONE ANSWER POSSIBLE]:

1. ❑ in traffic
2. ❑ while engaging in sports
3. ❑ during paid work
4. ❑ as a result of violence
5. ❑ intentional self-wounding
6. ❑ in a different way

97. ❑ d.k. 98. ❑ n.a. 99. ❑ N/A

ongev3 – Who treated you for this [MORE THAN ONE ANSWER POSSIBLE]?

1. ❑ general practitioner
2. ❑ physical therapist
3. ❑ emergency ward of a hospital
4. ❑ specialist, polyclinic (by appointment)
5. ❑ admitted to hospital
6. ❑ other

97. ❑ d.k. 98. ❑ n.a. 99. ❑ N/A

ongev4 – Which part of your body suffered the most serious injury [INT. ONLY ONE ANSWER POSSIBLE]?

1. ❑ head
2. ❑ neck
3. ❑ arm (including shoulder, collarbone)
4. ❑ torso (including back)
5. ❑ leg (including hip)

97. ❑ d.k. 98. ❑ n.a. 99. ❑ N/A

ongev5 – What is the nature of the most serious injury to this body part? [ONLY ONE ANSWER POSSIBLE]

1. ❑ open wound
2. ❑ bruise, contusion
3. ❑ sprain, strain, twist
4. ❑ broken bone, fracture
5. ❑ other injury

97. ❑ d.k. 98. ❑ n.a. 99. ❑ N/A

ACTIVITIES IN DAY-TO-DAY LIFE

*Int. Only ask the following questions (adl1 to adl3) if the respondent is* ***55*** *years of age or older (see flap)*

[INT. SHOW CARD 8]

adl1 - I am now going to mention a few acts that are difficult for some people. Would you please indicate each time on the basis of this card whether you can do them without difficulty, with some difficulty, with great difficulty or only with help from others? [INT. READ OUT AND WRITE ANSWERS IN TABLE 8]

TABLE 8 Activities in day-to-day life

|  | without difficulty | with some difficulty | with great difficulty | only with help from others |  |
| --- | --- | --- | --- | --- | --- |
| 1. eating and drinking | 1. ❑ | 2. ❑ | 3. ❑ | 4. ❑ |  d.k.  n.a.  N/A |
| 1. Sitting down on and getting up from a chair | 1. ❑ | 2. ❑ | 3. ❑ | 4. ❑ |  d.k.  n.a.  N/A |
| 1. Getting into or out of bed | 1. ❑ | 2. ❑ | 3. ❑ | 4. ❑ |  d.k.  n.a.  N/A |
| 1. Getting dressed or undressed | 1. ❑ | 2. ❑ | 3. ❑ | 4. ❑ |  d.k.  n.a.  N/A |
| 1. Going to another room on the same floor. | 1. ❑ | 2. ❑ | 3. ❑ | 4. ❑ |  d.k.  n.a.  N/A |
| 1. Going up or down the stairs. | 1. ❑ | 2. ❑ | 3. ❑ | 4. ❑ |  d.k.  n.a.  N/A |
| 1. leaving and entering the house | 1. ❑ | 2. ❑ | 3. ❑ | 4. ❑ |  d.k.  n.a.  N/A |
| 1. moving about outside the house | 1. ❑ | 2. ❑ | 3. ❑ | 4. ❑ |  d.k.  n.a.  N/A |
| 1. washing your face and hands | 1. ❑ | 2. ❑ | 3. ❑ | 4. ❑ |  d.k.  n.a.  N/A |
| 1. Washing your entire body | 1. ❑ | 2. ❑ | 3. ❑ | 4. ❑ |  d.k.  n.a.  N/A |

adl2 – Do you at present receive any help with your personal care? With personal care we mean one of the following activities: washing, bathing, taking a shower, getting dressed, going to the toilet, getting up and sitting down.

1. ❑ yes 🡪 go to **adl3**
2. ❑ no 🡪 go to **text-hda1**

97. ❑ d.k. 98. ❑ n.a. 99. ❑ N/A

adl3 – Who provides this help for you? [INT.: mORE THAN ONE answer possible, Do NOT READ OUT!!]

1. ❑ my partner
2. ❑ child living at home
3. ❑ another person living at home
4. ❑ Child living away from home
5. ❑ other relatives not living at home
6. ❑ neighbours, friends or acquaintances
7. ❑ volunteers
8. ❑ home care/nursing services
9. ❑ paid help / private help
10. ❑ someone else

97. ❑ d.k. 98. ❑ n.a. 99. ❑ N/A

###### DAILY HOUSEHOLD ACTIVITIES

*Int. Only ask the following questions (hda1a to hda6b) if the respondent is* ***55*** *years of age or older*

[INT. SHOW CARD 9]

text-hda1 - I will now mention some household activities that some people find difficult to perform. Could you please indicate whether you can do these things without difficulty, with difficulty or not at all. If you never perform the activity, you can also specify this.

hda1a – Keeping the house clean.

1. ❑ Without difficulty 🡪 go to **hda2a**
2. ❑ With difficulty 🡪 go to **hda2a**
3. ❑ Cannot do it 🡪 go to **hda2a**
4. ❑ Never do it 🡪 go to **hda1b**

97. ❑ d.k. 98. ❑ n.a. 99. ❑ N/A

hda1b – Could you do it?

1. ❑ yes
2. ❑ no

97. ❑ d.k. 98. ❑ n.a. 99. ❑ N/A

hda2a – Preparing hot meals

1. ❑ Without difficulty 🡪 go to **hda3a**
2. ❑ With difficulty 🡪 go to **hda3a**
3. ❑ Cannot do it 🡪 go to **hda3a**
4. ❑ Never do it 🡪 go to **hda2b**

97. ❑ d.k. 98. ❑ n.a. 99. ❑ N/A

hda2b – Could you do it?

1. ❑ yes
2. ❑ no

97. ❑ d.k. 98. ❑ n.a. 99. ❑ N/A

hda3a – Doing the shopping.

1. ❑ Without difficulty 🡪 go to **text-hda2**
2. ❑ With difficulty 🡪 go to **text-hda2**
3. ❑ Cannot do it 🡪 go to **text-hda2**
4. ❑ Never do it 🡪 go to **hda3b**

97. ❑ d.k. 98. ❑ n.a. 99. ❑ N/A

hda3b – Could you do it?

1. ❑ yes
2. ❑ no

97. ❑ d.k. 98. ❑ n.a. 99. ❑ N/A

*int. If the respondent has indicated that he/she has trouble with this or is unable to do this 🡪 then go on to the hda questions, otherwise go to the next block (tekst-voe1)*

**text-hda2** – I am now going to ask whether you receive help with several household tasks.

*INT. Only ask the following question if the respondent indicated that he/she has difficulty with* ***housecleaning****, or cannot do this (see questions hda****1****a and hda****1****b)*

hda4a – Is anyone helping you with the house cleaning at present?

1. ❑ yes 🡪 go to **hda4b**
2. ❑ no 🡪 go to **hda5a**

97. ❑ d.k. 98. ❑ n.a. 99. ❑ N/A

hda4b – From whom are you receiving this help? [INT.: MORE THAN ONE ANSWER possible, DO NOT READ OUT!]

1. ❑ partner
2. ❑ child living at home
3. ❑ another person living at home
4. ❑ child not living at home
5. ❑ other family not living at home
6. ❑ neighbours, friends or acquaintances
7. ❑ volunteers
8. ❑ home care
9. ❑ paid help / private help
10. ❑ someone else

97. ❑ d.k. 98. ❑ n.a. 99. ❑ N/A

*INT. Only ask the following question if the respondent indicated that he/she has difficulty in* ***preparing hot meals****, or cannot do this (see questions hda****2****a and hda****2****b).*

hda5a – Is anyone helping you to prepare hot meals at present?

1. ❑ yes 🡪 go to **hda5b**
2. ❑ no 🡪 go to **hda6a**

97. ❑ d.k. 98. ❑ n.a. 99. ❑ N/A

hda5b – From whom are you receiving this help? [INT.: MORE THAN ONE ANSWER possible, DO NOT READ OUT!]

1. ❑ partner
2. ❑ child living at home
3. ❑ other person living at home
4. ❑ child not living at home
5. ❑ other family not living at home
6. ❑ neighbours, friends or acquaintances
7. ❑ volunteers
8. ❑ home care
9. ❑ paid help / private help
10. ❑ someone else
11. ❑ home delivery of meals (meals-on-wheels)
12. ❑ open table (community centre, nursing home, centre for the elderly)

97. ❑ d.k. 98. ❑ n.a. 99. ❑ N/A

*INT. Only ask the following question if the respondent indicated that he/she had difficulty with* ***going shopping****, or cannot do this (see questions hda3a and hda3b)*

hda6a – Is anyone helping you with the shopping at present?

1. ❑ yes 🡪 go to **hda6b**
2. ❑ no 🡪 go to the next block, **text-voe1**

97. ❑ d.k. 98. ❑ n.a. 99. ❑ N/A

hda6b – From whom are you receiving this help? [INT.: MORE THAN ONE ANSWER possible, DO NOT READ OUT!]

1. ❑ partner
2. ❑ child living at home
3. ❑ another person living at home
4. ❑ child not living at home
5. ❑ other family not living at home
6. ❑ neighbours, friends or acquaintances
7. ❑ volunteers
8. ❑ home care
9. ❑ paid help / private help
10. ❑ someone else
11. ❑ odd job service, neighbourhood assistance, shopping assistance, volunteer organisations
12. ❑ shopping bus/ shopping service

97. ❑ d.k. 98. ❑ n.a. 99. ❑ N/A

###### NUTRITION

tekst-voe1: The next questions are about nutrition and things related to this.

bmi1 – How much do you weigh (without clothing)?

(if you are pregnant, we would like to know your weight from before the pregnancy)

|  |  |  | kilos | 97. ❑ d.k. 98. ❑ n.a. 99. ❑ N/A |
| --- | --- | --- | --- | --- |

bmi2 – How tall are you (without shoes)?

|  |  |  | centimetres | 97. ❑ d.k. 98. ❑ n.a. 99. ❑ N/A |
| --- | --- | --- | --- | --- |

##### Int. Only ask the next question if the respondent is a woman

bmi3 – Are you now pregnant?

1. ❑ yes 🡪 go to **bmi4**
2. ❑ no 🡪 go to **tekst-voe2**

97. ❑ d.k. 98. ❑ n.a. 99. ❑ N/A

bmi4 – How many weeks pregnant are you now?

|  |  |  | 97. ❑ d.k. 98. ❑ n.a. 99. ❑ N/A |
| --- | --- | --- | --- |

tekst-voe2: Now there are some questions about your eating pattern.

voe1a/b – How many days a week do you usually eat cooked or fried vegetables, salad, or raw vegetables?

Potatoes do not count as vegetables. Vegetables in one-pan meals (such as Dutch-style mashed vegetable dishes) do count, but a leaf of lettuce on a salad roll does not count. [🡪TABLE VOE1]

voe1c/d – On days on which you eat vegetables, salad, or raw vegetables, how many serving spoons do you usually eat? One serving spoon is about 50 grammes. [🡪TABLE VOE2]

voe2a/b – How many days a week do you usually eat fruit or drink fruit juice? [🡪TABLE VOE1]

voe2c – On the days on which you eat fruit, how many pieces do you eat? 1 piece of fruit, for example, is a medium-size apple or 2 mandarins. A handful of small fruit, such as cherries, can be counted as 1 piece. [🡪TABLE VOE2]

voed2d – On days on which you drink fruit juice, how many glasses of fruit juice (freshly squeezed or from a carton) do you drink then? [🡪TABLE VOE2]

| TABLE VOE1 | number of **days per week** | | | | | | | |  |
| --- | --- | --- | --- | --- | --- | --- | --- | --- | --- |
|  | Less than 1 | 1 | 2 | 3 | 4 | 5 | 6 | 7 | 97. 98. |
| 1a – cooked/fried vegetables | 0. ❑ | 1. ❑ | 2. ❑ | 3. ❑ | 4. ❑ | 5. ❑ | 6. ❑ | 7. ❑ | ❑ w.n ❑ n.a. |
| 1b - salad/raw vegetables | 0. ❑ | 1. ❑ | 2. ❑ | 3. ❑ | 4. ❑ | 5. ❑ | 6. ❑ | 7. ❑ | ❑ w.n ❑ n.a. |
| 2a - fruit | 0. ❑ | 1. ❑ | 2. ❑ | 3. ❑ | 4. ❑ | 5. ❑ | 6. ❑ | 7. ❑ | ❑ w.n ❑ n.a. |
| 2b – fruit juice (freshly squeezed or from a carton) | 0. ❑ | 1. ❑ | 2. ❑ | 3. ❑ | 4. ❑ | 5. ❑ | 6. ❑ | 7. ❑ | ❑ w.n ❑ n.a. |

| TABLE VOE2 | number **per day** | | | | | | |  |
| --- | --- | --- | --- | --- | --- | --- | --- | --- |
|  | N/A | 1 | 2 | 3 | 4 | 5 | More than 5 | 97. 98. |
| 1c – cooked/fried vegetables **(serving spoons)** | 99. ❑ | 1. ❑ | 2. ❑ | 3. ❑ | 4. ❑ | 5. ❑ | 6. ❑ | ❑ w.n ❑ n.a. |
| 1d - salad/raw vegetables **(serving spoons)** | 99. ❑ | 1. ❑ | 2. ❑ | 3. ❑ | 4. ❑ | 5. ❑ | 6. ❑ | ❑ w.n ❑ n.a. |
| 2c - fruit **(pieces)** | 99. ❑ | 1. ❑ | 2. ❑ | 3. ❑ | 4. ❑ | 5. ❑ | 6. ❑ | ❑ w.n ❑ n.a. |
| 2d – fruit juice **(glasses)** | 99. ❑ | 1. ❑ | 2. ❑ | 3. ❑ | 4. ❑ | 5. ❑ | 6. ❑ | ❑ w.n ❑ n.a. |

voe3 – How many days a week do you usually have breakfast? Breakfast drink, breakfast bar, muesli, etc. also count as breakfast.

|  | Number of **days per week** | | | | | | | |  |
| --- | --- | --- | --- | --- | --- | --- | --- | --- | --- |
|  | Less than 1 | 1 | 2 | 3 | 4 | 5 | 6 | 7 | 97. 98. 99. |
| breakfast | 0. ❑ | 1. ❑ | 2. ❑ | 3. ❑ | 4. ❑ | 5. ❑ | 6. ❑ | 7. ❑ | ❑ w.n ❑ n.a. ❑ N/A |

voe4 – When did you last eat today? [INT. FILL IN TIME]

| 1. ❑  |  |  | : |  |  |
| --- | --- | --- | --- | --- | --- |

1. ❑ I have not eaten at all yet today 🡪 go to the next block, **text-voe3**

97. ❑ d.k. 98. ❑ n.a. 99. ❑ N/A

voe5 – What kind of meal did you eat then? [INT. Assist if necessary]

1. ❑ breakfast
2. ❑ hot lunch
3. ❑ cold lunch
4. ❑ hot dinner
5. ❑ cold dinner
6. ❑ a snack

97. ❑ d.k. 98. ❑ n.a. 99. ❑ N/A

tekst-voe3: With the next questions I would like to focus on what you usually eat and drink. Think about what you ate and drank in the past month here.

voe6a – How many days a week do you usually drink milk or buttermilk? [🡪TABLE VOE3]

voe6b – On a day on which you drink milk or buttermilk, how many glasses do you usually drink per day? [🡪TBL VOE4]

voe6c – What type of milk do you usually drink? [INT. ONLY ONE ANSWER IS POSSIBLE]

1. ❑ not applicable
2. ❑ whole milk
3. ❑ semi-skimmed milk
4. ❑ skimmed milk
5. ❑ buttermilk
6. ❑ varies

97. ❑ d.k. 98. ❑ n.a. 99. ❑ N/A

voe7a – How many days a week do you usually drink chocolate? [🡪TABLE VOE3]

voe7b – On a day on which you drink chocolate, how many glasses do you usually drink per day? [🡪TBL VOE4]

voe7c – What type of chocolate do you usually drink? [INT. ONLY ONE ANSWER IS POSSIBLE]

1. ❑ not applicable
2. ❑ whole chocolate
3. ❑ semi-skimmed chocolate
4. ❑ skimmed chocolate
5. ❑ varies

97. ❑ d.k. 98. ❑ n.a.

voe8a – How many days a week do you usually eat yoghurt, fruit yoghurt or biogarde yoghurt? [🡪TABLE VOE3]

voe8b – On a day on which you eat yoghurt, fruit yoghurt or biogarde yoghurt, how many bowls do you usually eat per day? [🡪TABLE VOE4]

voe8c – What type of yoghurt, fruit yoghurt, or biogarde yoghurt do you usually eat? [INT. ONLY ONE ANSWER IS POSSIBLE]

1. ❑ not applicable
2. ❑ whole yoghurt
3. ❑ semi-skimmed yoghurt
4. ❑ skimmed yoghurt
5. ❑ varies

97. ❑ d.k. 98. ❑ n.a.

voe9a – How many days a week do you usually eat (liquid) pudding or porridge? [🡪TABLE VOE3]

voed9b- On a day on which you eat (liquid) pudding or porridge, how many bowls do you usually eat per day? [🡪 VOE4]

| TABLE VOE3 | number of **days per week** | | | | | | | |  |
| --- | --- | --- | --- | --- | --- | --- | --- | --- | --- |
|  | Never or less than 1 | 1 | 2 | 3 | 4 | 5 | 6 | 7 | 97. 98. |
| 6a. milk or buttermilk | 0. ❑ | 1. ❑ | 2. ❑ | 3. ❑ | 4. ❑ | 5. ❑ | 6. ❑ | 7. ❑ | ❑ w.n ❑ n.a. |
| 7a. chocolate | 0. ❑ | 1. ❑ | 2. ❑ | 3. ❑ | 4. ❑ | 5. ❑ | 6. ❑ | 7. ❑ | ❑ w.n ❑ n.a. |
| 8a. yoghurt, fruit yoghurt, biogarde yoghurt | 0. ❑ | 1. ❑ | 2. ❑ | 3. ❑ | 4. ❑ | 5. ❑ | 6. ❑ | 7. ❑ | ❑ w.n ❑ n.a. |
| 9a. (liquid) pudding, porridge | 0. ❑ | 1. ❑ | 2. ❑ | 3. ❑ | 4. ❑ | 5. ❑ | 6. ❑ | 7. ❑ | ❑ w.n ❑ n.a. |

| TABLE VOE4 | number of **glasses/bowls** per day | | | | | |  |
| --- | --- | --- | --- | --- | --- | --- | --- |
|  | N/A | 1 | 2 | 3 | 4 | 5 or more | 97. 98. |
| 6b. milk or buttermilk | 99. ❑ | 1. ❑ | 2. ❑ | 3. ❑ | 4. ❑ | 5. ❑ | ❑ w.n ❑ n.a. |
| 7b. chocolate | 99. ❑ | 1. ❑ | 2. ❑ | 3. ❑ | 4. ❑ | 5. ❑ | ❑ w.n ❑ n.a. |
| 8b. yoghurt, fruit yoghurt, biogarde yoghurt | 99. ❑ | 1. ❑ | 2. ❑ | 3. ❑ | 4. ❑ | 5. ❑ | ❑ w.n ❑ n.a. |
| 9b. (liquid) pudding, porridge | 99. ❑ | 1. ❑ | 2. ❑ | 3. ❑ | 4. ❑ | 5. ❑ | ❑ w.n ❑ n.a. |

voe10 – How many slices of bread, rolls, crackers, or biscuit rusks do you usually eat per day? [🡪TABLE VOE5]

voe11 – How many slices of bread with cheese do you usually have? [🡪TABLE VOE5]

voe11b – What type of cheese do you usually have on your bread? [INT. ONLY ONE ANSWER IS POSSIBLE]

1. ❑ not applicable
2. ❑ non-Dutch cheeses such as Brie, Camembert, Paturain, etc.
3. ❑ Gouda cheese, Edam or other 40+ and 48+ types
4. ❑ 20+ and 30+ types, such as Westlite, Milner, Linera etc.

97. ❑ d.k. 98. ❑ n.a.

voe12 – How many times do you usually have meat on your bread? [🡪TABLE VOE5]

voe12b – What type of meat do you usually put on your bread (describe as precisely as possible)?

1. ❑ not applicable
2. ❑ ……………………………………………………………….
3. ❑ ……………………………………………………………….
4. ❑ ……………………………………………………………….

97. ❑ d.k. 98. ❑ n.a.

voe13 – How many slices of bread do you usually have with chocolate sprinkles, chocolate flakes or chocolate paste? [🡪TABLE VOE5]

| TABLE VOE5 | number of **slices per day** | | | | | | | | | |  |
| --- | --- | --- | --- | --- | --- | --- | --- | --- | --- | --- | --- |
|  | none | 1 | 2 | 3 | 4 | 5 | 6 | 7 | 8 | 9 or more | 97. 98. |
| 10. slices of bread etc. | 0. ❑ | 1. ❑ | 2. ❑ | 3. ❑ | 4. ❑ | 5. ❑ | 6. ❑ | 7. ❑ | 8. ❑ | 9. ❑ | ❑ w.n ❑ n.a. |
| 11. with cheese | 0. ❑ | 1. ❑ | 2. ❑ | 3. ❑ | 4. ❑ | 5. ❑ | 6. ❑ | 7. ❑ | 8. ❑ | 9. ❑ | ❑ w.n ❑ n.a. |
| 12. with meat | 0. ❑ | 1. ❑ | 2. ❑ | 3. ❑ | 4. ❑ | 5. ❑ | 6. ❑ | 7. ❑ | 8. ❑ | 9. ❑ | ❑ w.n ❑ n.a. |
| 1. with chocolate sprinkles etc. | 0. ❑ | 1. ❑ | 2. ❑ | 3. ❑ | 4. ❑ | 5. ❑ | 6. ❑ | 7. ❑ | 8. ❑ | 9. ❑ | ❑ w.n ❑ n.a. |

voe14 – What type of butter do you usually put on your bread? [INT. ONLY ONE ANSWER IS POSSIBLE]

1. ❑ no butter
2. ❑ butter
3. ❑ margarine
4. ❑ diet margarine
5. ❑ low-fat margarine, low-fat butter, yoghurt-based butter
6. ❑ diet low-fat margarine
7. ❑ Linera 25% or Twenty Four
8. ❑ varies

97. ❑ d.k. 98. ❑ n.a.

voe15 – How often do you eat smoked sausage, fresh sausage or other sausages with your hot meal? [🡪TABLE VOE6]

voe16 – How often do you have beef-and-pork mince with your hot meal? [🡪TABLE VOE6]

voe17 – How often do you have slices of bacon with your hot meal? [🡪TABLE VOE6]

voe18 – How often do you have cheese (grated, diced in your food, or cheese soufflé) with your hot meal? [🡪VOE6]

voe19 – How often do you have gravy with your hot meal? [TABLE VOE6]

| TABLE VOE6 | number of times | | | | | | |  |
| --- | --- | --- | --- | --- | --- | --- | --- | --- |
| **with** the hot meal | Never or less than once a month | Once a month | Once in 3 weeks | Once in 2 weeks | Once a week | Twice a week | 3 times or more often per week | 97. 98. |
| 15. smoked sausage etc. | 0. ❑ | 1. ❑ | 2. ❑ | 3. ❑ | 4. ❑ | 5. ❑ | 6. ❑ | ❑ w.n ❑ n.a. |
| 16. beef and pork mince | 0. ❑ | 1. ❑ | 2. ❑ | 3. ❑ | 4. ❑ | 5. ❑ | 6. ❑ | ❑ w.n ❑ n.a. |
| 17. slices of bacon | 0. ❑ | 1. ❑ | 2. ❑ | 3. ❑ | 4. ❑ | 5. ❑ | 6. ❑ | ❑ w.n ❑ n.a. |
| 18. cheese | 0. ❑ | 1. ❑ | 2. ❑ | 3. ❑ | 4. ❑ | 5. ❑ | 6. ❑ | ❑ w.n ❑ n.a. |
| 19. gravy | 0. ❑ | 1. ❑ | 2. ❑ | 3. ❑ | 4. ❑ | 5. ❑ | 6. ❑ | ❑ w.n ❑ n.a. |

voe19b – How many spoonfuls do you take when you have gravy?

|  | number of **spoonfuls** | | | | | | |  |
| --- | --- | --- | --- | --- | --- | --- | --- | --- |
|  | N/A | 1 | 2 | 3 | 4 | 5 | 6 or more | 97. 98. |
| 19b. gravy | 99. ❑ | 1. ❑ | 2. ❑ | 3. ❑ | 4. ❑ | 5. ❑ | 6. ❑ | ❑ w.n ❑ n.a. |

voe19c – What type of spoon do you use to ladle out the gravy?

1. ❑ not applicable
2. ❑ soupspoon
3. ❑ small gravy-spoon
4. ❑ large gravy-spoon

97. ❑ d.k. 98. ❑ n.a.

voe20 – How often do you have snacks such as chips, croquettes, potato salads, etc?

|  | number of times | | | | | | |  |
| --- | --- | --- | --- | --- | --- | --- | --- | --- |
| as a **side dish** | never or less than once a month | once a month | once in 3 weeks | once in 2 weeks | once a week | twice a week | 3 times a week or more often | 97. 98. |
| 20. snacks (chips etc.) | 0. ❑ | 1. ❑ | 2. ❑ | 3. ❑ | 4. ❑ | 5. ❑ | 6. ❑ | ❑ w.n ❑ n.a. |

voe21 – How often do you have peanuts or nuts between times? [🡪TABLE VOE7]

voe22 – How often do you have crisps, diced cheese, non-Dutch cheese or sausage between times? [🡪TABLE VOE7]

voe23 – How often do you have pastries or cake between times? [🡪TABLE VOE7]

voe24 – How often do you have candy bars such as Nuts, Mars, Snickers etc. between times? [🡪TABLE VOE7]

voe25 – How often do you have chocolate between times? [🡪TABLE VOE7]

voe26 – How often do you eat biscuits or raisin biscuits between times? [🡪TABLE VOE7]

voe26a – How many biscuits do you usually eat on a day when you eat biscuits?

1. ❑ not applicable
2. ❑ 1 per day
3. ❑ 2 per day
4. ❑ 3 per day
5. ❑ 4 per day
6. ❑ 5 per day
7. ❑ 6 per day
8. ❑ 7 or more per day

97. ❑ d.k. 98. ❑ n.a.

voe27 – How often do you eat other cookies between times? [🡪TABLE VOE7]

voe27a – How many cookies do you usually eat on a day when you eat cookies?

1. ❑ not applicable
2. ❑ 1 per day
3. ❑ 2 per day
4. ❑ 3 per day
5. ❑ 4 per day
6. ❑ 5 per day
7. ❑ 6 per day
8. ❑ 7 or more per day

97. ❑ d.k. 98. ❑ n.a.

| TABLE VOE7 | number of **days per week** | | | | | | | |  |
| --- | --- | --- | --- | --- | --- | --- | --- | --- | --- |
| **between times** | never or less than 1 day per week | 1 day  per week | 2 days per week | 3 days per week | 4 days per week | 5 days per week | 6 days per week | 7 days per week | 97. 98. |
| 21. peanuts or nuts | 0. ❑ | 1. ❑ | 2. ❑ | 3. ❑ | 4. ❑ | 5. ❑ | 6. ❑ | 7. ❑ | ❑ w.n ❑ n.a. |
| 22. crisps, cheese, etc. | 0. ❑ | 1. ❑ | 2. ❑ | 3. ❑ | 4. ❑ | 5. ❑ | 6. ❑ | 7. ❑ | ❑ w.n ❑ n.a. |
| 23. pastry, cake | 0. ❑ | 1. ❑ | 2. ❑ | 3. ❑ | 4. ❑ | 5. ❑ | 6. ❑ | 7. ❑ | ❑ w.n ❑ n.a. |
| 24. candy bars | 0. ❑ | 1. ❑ | 2. ❑ | 3. ❑ | 4. ❑ | 5. ❑ | 6. ❑ | 7. ❑ | ❑ w.n ❑ n.a. |
| 25. chocolate | 0. ❑ | 1. ❑ | 2. ❑ | 3. ❑ | 4. ❑ | 5. ❑ | 6. ❑ | 7. ❑ | ❑ w.n ❑ n.a. |
| 26. (raisin) biscuit | 0. ❑ | 1. ❑ | 2. ❑ | 3. ❑ | 4. ❑ | 5. ❑ | 6. ❑ | 7. ❑ | ❑ w.n ❑ n.a. |
| 27. other cookies | 0. ❑ | 1. ❑ | 2. ❑ | 3. ❑ | 4. ❑ | 5. ❑ | 6. ❑ | 7. ❑ | ❑ w.n ❑ n.a. |

USE OF ALCOHOL, TOBACCO

tekst-alc: The next questions are about the use of alcohol and tobacco.

[INT. TOON KAART 10]

alc1 – Please indicate what types of alcoholic beverages you drunk in the past 12 months. Which others? [INT. SEVERAL ANSWERS POSSIBLE]

1. ❑ beer (no mildly or non-alcoholic beer)
2. ❑ wine, sherry, port, vermouth
3. ❑ liqueur, advocaat, blackcurrant jenever, lemon jenever 🡪 go to **alc2a**
4. ❑ jenever, brandy, vieux, rum, cognac, whisky, vodka or other spirits
5. ❑ alcoholic beverage mixed with soft drink or fruit juice (e.g. breezers, shooters)
6. ❑ I used to drink, but I have not drunk any alcoholic beverages in the past 12 months 🡪 **rook1**
7. ❑ I have never drunk any alcoholic beverages 🡪 go to **rook1**

97. ❑ d.k. 98. ❑ n.a. 99. ❑ N/A

alc2a – On how many of the four weekdays (meaning Monday through Thursday) do you drink alcoholic beverages on average?

1. ❑ 4 days
2. ❑ 3 days
3. ❑ 2 days 🡪 go to **alc2b**
4. ❑ 1 day
5. ❑ less than 1 day
6. ❑ I never drink on weekdays 🡪 go to **alc3a**

97. ❑ d.k. 98. ❑ n.a. 99. ❑ N/A

alc2b – When drinking alcoholic beverages on such a weekday, how many glasses do you drink on average? This still concerns Monday through Friday. The type of glass that is usual for that drink is meant. Count 1.5 glasses for a can or a bottle. So two bottles is 3 glasses (round up half glasses). [INT.: FILL IN NUMBER OF GLASSES:]

|  |  | glasses | 97. ❑ d.k. 98. ❑ n.a. 99. ❑ N/A |
| --- | --- | --- | --- |

alc3a – On average, on how many of the three weekend days (meaning Friday through Sunday) do you drink alcoholic beverages?

1. ❑ 3 days
2. ❑ 2 days
3. ❑ 1 day 🡪 go to **alc3b**
4. ❑ less than 1 day
5. ❑ I never drink at the weekend 🡪 go to **alc4**

97. ❑ d.k. 98. ❑ n.a. 99. ❑ N/A

alc3b – When drinking alcoholic beverages on such weekend days, how many glasses do you drink on average? This still concerns Fridays through Sundays. The type of glass that is usual for that drink is meant. Count 1.5 glasses for a can or a bottle. So two bottles is 3 glasses. (round up half glasses). [INT.: FILL IN NUMBER OF GLASSES:]

|  |  | glasses | 97. ❑ d.k. 98. ❑ n.a. 99. ❑ N/A |
| --- | --- | --- | --- |

alc4 – How often have you drunk 4 or more glasses of alcoholic beverages on one day in the past six months and how often did you drink 6 glasses or more on one day in the same period? [INT.: IF THE RESPONDENT ANSWERS, FOR EXAMPLE: 18 TIMES, THEN CALCULATE: COMES DOWN TO ABOUT 3 TIMES A MONTH]

|  | every day | 5-6 times a week | 3-4 times a week | 1-2 times a week | 1-3 times a month | 3-5 times per  half year | 1-2 times per  half year | never | 97. 98. 99. |
| --- | --- | --- | --- | --- | --- | --- | --- | --- | --- |
| 4 or more glasses | 1. ❑ | 2. ❑ | 3. ❑ | 4. ❑ | 5. ❑ | 6. ❑ | 7. ❑ | 8. ❑ | ❑ w.n ❑ n.a. ❑ N/A |
| 6 or more glasses | 1. ❑ | 2. ❑ | 3. ❑ | 4. ❑ | 5. ❑ | 6. ❑ | 7. ❑ | 8. ❑ | ❑ w.n ❑ n.a. ❑ N/A |

rook1 – Do you smoke (now and then)?

1. ❑ yes 🡪 go to **rook2**
2. ❑ no, but I used to 🡪 go to **drug1**
3. ❑ no, I have never smoked 🡪 go to **drug1**

97. ❑ d.k. 98. ❑ n.a. 99. ❑ N/A

rook2 - What do you smoke and how much?

|  | ± |  |  | cigarettes (from pack or self-rolled) a **day** |
| --- | --- | --- | --- | --- |
|  | ± |  |  | cigars a **week** |
|  | ± |  |  | pack(s) of pipe tobacco (50 grams) a **week** |
| 97. ❑ d.k. 98. ❑ n.a. 99. ❑ N/A | | | | |

drug1 – Now a number of questions follow about the use of cannabis. Have you ever used cannabis (hashish or ‘pot’ (marihuana))?

1. ❑ yes 🡪 go to **drug2**
2. ❑ no 🡪 go to **hdu1**

97. ❑ d.k. 98. ❑ n.a. 99. ❑ N/A

drug2 – Have you used cannabis (hashish or ‘pot’ (marihuana)) in the last 12 months?

1. ❑ yes 🡪 go to **drug3**
2. ❑ no 🡪 go to **hdu1**

97. ❑ d.k. 98. ❑ n.a. 99. ❑ N/A

drug3 – How often have you used cannabis (hashish or ‘pot’ (marihuana)) in the last 4 weeks?

1. ❑ 5 to 7 times a week
2. ❑ 3 to 4 times a week
3. ❑ once or twice a week
4. ❑ 1 to 3 times a month
5. ❑ 0 times

97. ❑ d.k. 98. ❑ n.a. 99. ❑ N/A

hdu1 – Have you ever taken hard drugs (hard drugs include cocaine, heroin or amphetamine (incl. XTC) )?

1. ❑ yes 🡪 go to **hdu2**
2. ❑ no 🡪 go to the next block, **tekst-bew**

97. ❑ d.k. 98. ❑ n.a. 99. ❑ N/A

hdu2 – How often did/do you take these hard drugs? [INT. ASSIST IF NECESSARY]

1. ❑ Rarely (less than once a month)
2. ❑ Sometimes (a few times a month, less than 3 times a week)
3. ❑ Regularly (at least 3 times a week on average)

97. ❑ d.k. 98. ❑ n.a. 99. ❑ N/A

hdu3 – Which hard drugs did/do you usually take?

1. ❑ Cocaine
2. ❑ Heroin
3. ❑ Combination of cocaine and heroin
4. ❑ XTC
5. ❑ Other amphetamines, not XTC
6. ❑ Other, namely…………………………………………………………………

97. ❑ d.k. 98. ❑ n.a. 99. ❑ N/A

hdu4 – Have you ever taken hard drugs intravenously?

1. ❑ No, I have never taken hard drugs intravenously 🡪 go to the next block, **tekst-bew**
2. ❑ Yes, the last time was in the past year 🡪 go to **hdu5**
3. ❑ Yes, the last time was not in the past year, but less than 5 years ago 🡪 go to **hdu5**
4. ❑ Yes, the last time was more than 5 years ago 🡪 go to the next block, **tekst-bew**

97. ❑ d.k. 98. ❑ n.a. 99. ❑ N/A

hdu5 – Have you ever borrowed injection material from other people?

1. ❑ yes 🡪 go to **hdu6**
2. ❑ no 🡪 go to the next block, **tekst-bew**

97. ❑ d.k. 98. ❑ n.a. 99. ❑ N/A

hdu6 – Were these people from a different population group than you?

1. ❑ Yes, they were mainly [check no more than 3]

❑ Dutch

❑ Surinamese

❑ Creole Surinamese

❑ Hindustani Surinamese

❑ Chinese Surinamese

❑ Javanese Surinamese

❑ Antillian

❑ Aruban

❑ Moroccan

❑ Berber

❑ Turkish

❑ Kurdish

❑ other, namely …………………………………………………

1. ❑ No

97. ❑ d.k. 98. ❑ n.a. 99. ❑ N/A

###### PHYSICAL EXERCISE

tekst-bew: Imagine a normal week in the past months. Please indicate how many days a week you engaged in the activities I am going to name. Please also indicate how much time you spent on this on average per day.

| Travelling to and from work/school | **a** | | | | | | | | | | **b** | | | | | | | | | | | | | | | | | | |  |
| --- | --- | --- | --- | --- | --- | --- | --- | --- | --- | --- | --- | --- | --- | --- | --- | --- | --- | --- | --- | --- | --- | --- | --- | --- | --- | --- | --- | --- | --- | --- |
|  | number of days  each *week* | | | | | | | | | | average time  each *day* | | | | | | | | | | | | | | | | | | | 97. 99. |
|  |  | | | | | | | | | |  | | | | | | | | | | | | | | | | | | |  |
| Bew01 – walking to/from work or school |  |  | | | days | | | | | |  | | | |  | | | | hrs | | |  | | |  | | | min | | d.k. ❑   N/A ❑ |
|  |  | | | | | | | | | |  | | | | | | | | | | | | | | | | | | |  |
| Bew02 – cycling to /from work or school |  |  | | | days | | | | | |  | | | |  | | | | hrs | | |  | | |  | | | min | | d.k. ❑   N/A ❑ |
|  |  | | | | | | | | | |  | | | | | | | | | | | | | | | | | | |  |
| Physical activity at work and school | Hours each *week* | | | | | | | | | | | | | | | | | | | | | | | | | | | | |  |
|  |  | | | | | | | | | | | | | | | | | | | | | | | | | | | | | d.k. ❑   N/A ❑ |
| Bew03 – Work requiring **light and moderate** effort |  | | | | | | |  |  | hrs | | | | | | | | | | | | | | | | | | | |
|  |  | | | | | | |  |  |  | | | | | | | | | | | | | | | | | | | |  |
| Bew04 – Work involving **heavy effort** |  | | | | | | |  |  | hrs | | | | | | | | | | | | | | | | | | | | d.k. ❑   N/A ❑ |
| (work done while walking, or in which heavy things |  | | | | | | | | | | | | | | | | | | | | | | | | | | | | |  |
| Have to be lifted regularly) |  | | | | | | | | | | | | | | | | | | | | | | | | | | | | |  |
| Household activities | **a** | | | | | | | | | | | **b** | | | | | | | | | | | | | | | | | |  |
|  | number of days  each *week* | | | | | | | | | | | Average time  each *day* | | | | | | | | | | | | | | | | | |  |
|  |  | | | | | | | | | | |  | | | | | | | | | | | | | | | | | | d.k. ❑   N/A ❑ |
| Bew05 – Housework requiring light and moderate effort |  |  | | | days | | | | | | |  |  | | | | | Hrs | | |  | | |  | | | min | | |
| (cooking, washing up, ironing etc.) |  | |  | | |  | | | | | |  | |  | | |  | | | |  | | |  | | |  | | |  |
|  |  | |  | | |  | | | | | |  | |  | | |  | | | |  | | |  | | |  | | | d.k. ❑   N/A ❑ |
| Bew06 – Housework requiring heavy effort |  |  | | | days | | | | | | |  |  | | | | | Hrs | | |  | | |  | | | min | | |
| (scrubbing the floor, walking with heavy packages) |  | | | | | | | | | | |  | | | | | | | | | | | | | | | | | |  |
|  |  | | | | | | | | | | |  | | | | | | | | | | | | | | | | | |  |
| Leisure time | **a** | | | | | | | | | | | **b** | | | | | | | | | | | | | | | | | |
|  | number of days  each *week* | | | | | | | | | | | Average time  each *day* | | | | | | | | | | | | | | | | | |
|  |  | | | | | | | | | | |  | | | | | | | | | | | | | | | | | |
| Bew07 – Walking |  | | |  | | | days | | | | |  | | | |  | | | | Hrs | | |  | | |  | | | min | d.k. ❑   N/A ❑ |
|  |  | | |  | | |  | | | | |  | | | |  | | | |  | | |  | | |  | | |  |
| Bew08 – Cycling |  | | |  | | | days | | | | |  | | | |  | | | | Hrs | | |  | | |  | | | min | d.k. ❑   N/A ❑ |
|  |  | | |  | | |  | | | | |  | | | |  | | | |  | | |  | | |  | | |  |
| Bew09 - Gardening |  | | |  | | | days | | | | |  | | | |  | | | | Hrs | | |  | | |  | | | min | d.k. ❑   N/A ❑ |
|  |  | | |  | | |  | | | | |  | | | |  | | | |  | | |  | | |  | | |  |
| Bew10 – Odd jobs/do-it-yourself jobs |  | | |  | | | days | | | | |  | | | |  | | | | Hrs | | |  | | |  | | | min | d.k. ❑   N/A ❑ |
|  |  | | |  | | |  | | | | |  | | | |  | | | |  | | |  | | |  | | |  |
| Bew11a - sport1, namely…………………………… |  | | |  | | | days | | | | |  | | | |  | | | | Hrs | | |  | | |  | | | min | d.k. ❑   N/A ❑ |
|  |  | | |  | | |  | | | | |  | | | |  | | | |  | | |  | | |  | | |  |
| Bew11b – sport2, namely…………………………… |  | | |  | | | days | | | | |  | | | |  | | | | Hrs | | |  | | |  | | | min | d.k. ❑   N/A ❑ |
|  |  | | |  | | |  | | | | |  | | | |  | | | |  | | |  | | |  | | |  |
| Bew11c – sport3, namely…………………………… |  | | |  | | | days | | | | |  | | | |  | | | | Hrs | | |  | | |  | | | min | d.k. ❑   N/A ❑ |
|  |  | | |  | | |  | | | | |  | | | |  | | | |  | | |  | | |  | | |  |
| Bew11d - sport4, namely…………………………… |  | | |  | | | days | | | | |  | | | |  | | | | Hrs | | |  | | |  | | | min | d.k. ❑   N/A ❑ |

ENVIRONMENT

Tekst-omg1: Now I would like to ask you some questions about your home and your environment

|  | very happy | | happy | | not happy, not unhappy | | unhappy | | very unhappy | | no opinion / don’t know | |
| --- | --- | --- | --- | --- | --- | --- | --- | --- | --- | --- | --- | --- |
| Omg1 - How happy are you with your home? | 1. ❑ | | 2. ❑ | | 3. ❑ | | 4. ❑ | | 5. ❑ | | 6. ❑ | |
| Omg2 - How happy are you with your environment? | 1. ❑ | | 2. ❑ | | 3. ❑ | | 4. ❑ | | 5. ❑ | | 6. ❑ | |
| Tekst-omg2: With the following questions we would like to find out how you experience your home and the neighbourhood where you live. What you are asked to do is to indicate to what extent each of the following statements applies to your living situation. | | | | | | | | | | | | |
|  | | applies perfectly | | applies | | Neutral | | does not apply | | does not apply at all | | no opinion / don’t know |
| Omg3 - There is much pollution, graffiti and vandalism in my neighbourhood. | | 1. ❑ | | 2. ❑ | | 3. ❑ | | 4. ❑ | | 5. ❑ | | 6. ❑ |
| omg4 - My neighbourhood is a social neighbourhood with a great sense of unity. | | 1. ❑ | | 2. ❑ | | 3. ❑ | | 4. ❑ | | 5. ❑ | | 6. ❑ |
| omg5 - The people living in my neighbourhood do much to be annoyed about. | | 1. ❑ | | 2. ❑ | | 3. ❑ | | 4. ❑ | | 5. ❑ | | 6. ❑ |
| omg6 - I am comfortable with the people in my neighbourhood. | | 1. ❑ | | 2. ❑ | | 3. ❑ | | 4. ❑ | | 5. ❑ | | 6. ❑ |
| omg7 - My neighbourhood is known for its high rate of burglaries. | | 1. ❑ | | 2. ❑ | | 3. ❑ | | 4. ❑ | | 5. ❑ | | 6. ❑ |
| omg8 - The people in the neighbourhood barely know each other. | | 1. ❑ | | 2. ❑ | | 3. ❑ | | 4. ❑ | | 5. ❑ | | 6. ❑ |
| omg9 - There are many green areas in my neighbourhood. | | 1. ❑ | | 2. ❑ | | 3. ❑ | | 4. ❑ | | 5. ❑ | | 6. ❑ |
| omg10 - There is much traffic in my neighbourhood. | | 1. ❑ | | 2. ❑ | | 3. ❑ | | 4. ❑ | | 5. ❑ | | 6. ❑ |
| omg11 - The lack of parking space is a great nuisance in my neighbourhood. | | 1. ❑ | | 2. ❑ | | 3. ❑ | | 4. ❑ | | 5. ❑ | | 6. ❑ |
| omg12 – The houses in my neighbourhood are built closely together. | | 1. ❑ | | 2. ❑ | | 3. ❑ | | 4. ❑ | | 5. ❑ | | 6. ❑ |
| omg13 - In my neighbourhood there are shops for daily needs close by. | | 1. ❑ | | 2. ❑ | | 3. ❑ | | 4. ❑ | | 5. ❑ | | 6. ❑ |
| omg14 - There are good schools and types of schools in the neighbourhood. | | 1. ❑ | | 2. ❑ | | 3. ❑ | | 4. ❑ | | 5. ❑ | | 6. ❑ |
| omg15 - There is much room to play for children and young people in my neighbourhood. | | 1. ❑ | | 2. ❑ | | 3. ❑ | | 4. ❑ | | 5. ❑ | | 6. ❑ |
| omg16 – My home is easy to get to by public transportation. | | 1. ❑ | | 2. ❑ | | 3. ❑ | | 4. ❑ | | 5. ❑ | | 6. ❑ |
| omg17 - It is easy to get to facilities etc. by bicycle from my home. | | 1. ❑ | | 2. ❑ | | 3. ❑ | | 4. ❑ | | 5. ❑ | | 6. ❑ |

omg18 – Do you want to move within 2 years?

1. ❑ certainly not 🡪 go to **omg20**
2. ❑ possibly, maybe
3. ❑ would like to, but can’t find anything
4. ❑ absolutely
5. ❑ I’ve already found another home/place to live.

97. ❑ d.k. 98. ❑ n.a. 99. ❑ N/A

omg19 – For what reason do you want to move? (several answers are possible)

1. ❑ health or need for care
2. ❑ studies
3. ❑ work
4. ❑ dissatisfied with present home
5. ❑ dissatisfied with the environment
6. ❑ other, namely.…………………………………………………

97. ❑ d.k. 98. ❑ n.a. 99. ❑ N/A

omg20 – Do you think your neighbourhood has improved or deteriorated in the past year?

1. ❑ improved
2. ❑ stayed the same
3. ❑ deteriorated

97. ❑ d.k. 98. ❑ n.a. 99. ❑ N/A

omg21 – Do you think that the neighbourhood you live in will improve or deteriorate in the coming year?

1. ❑ improve
2. ❑ stay the same
3. ❑ deteriorate

97. ❑ d.k. 98. ❑ n.a. 99. ❑ N/A

omg22 – What do you think is the biggest problem in your neighbourhood? [INT. ONLY ONE ANSWER IS POSSIBLE]

1. ❑ noise pollution
2. ❑ soil contamination
3. ❑ rubbish in the streets and dogs’ messes
4. ❑ odour nuisance
5. ❑ hazard posed by industrial companies
6. ❑ water pollution
7. ❑ air pollution
8. ❑ encroachments on green areas (less green in and around the city)
9. ❑ burden on the environment by traffic
10. ❑ other, namely.…………………………………………………

97. ❑ d.k. 98. ❑ n.a. 99. ❑ N/A

TRAVEL

reis1 – The following questions are about travels you may have made to countries in the tropics or subtropics, the Balkan, former Soviet Union, Asia, Turkey, Morocco, Caribbean, Africa. Did you visit one or more of these countries in the past year ? [INT: ONLY THE COUNTRY OF DESTINATION OR COUNTRIES VISITED DURING A TOUR ARE OF INTEREST, NOT COUNTRIES WHICH WERE VISITED DURING A DIRECT TRIP THERE]

1. ❑ yes 🡪 go to **reis2**
2. ❑ no 🡪 go to the next block, **acc1**

97. ❑ d.k. 98. ❑ n.a. 99. ❑ N/A

reis2 – Which country/countries did you visit in the past year? [INT. SEVERAL ANSWERS ARE POSSIBLE]

1. ❑ Surinam
2. ❑ Netherlands Antilles
3. ❑ Aruba
4. ❑ Turkey
5. ❑ Morocco
6. ❑ Africa, namely……………………………………………………..
7. ❑ Balkan
8. ❑ Soviet Union
9. ❑ Azia
10. ❑ other, namely…………………………………………………….

97. ❑ d.k. 98. ❑ n.a. 99. ❑ N/A

reis3 – Did you have sexual contact with people living in these countries in the past year?

1. ❑ yes
2. ❑ no

97. ❑ d.k. 98. ❑ n.a. 99. ❑ N/A

reis4 – Did you get any “holiday shots” before the trip to the above-mentioned country/countries? [Int. IF THE PERSON DOES NOT HAVE A (YELLOW) VACCINATION BOOKLET, THEN HE OR SHE WAS NOT VACCINATED]

1. ❑ yes
2. ❑ no

97. ❑ d.k. 98. ❑ n.a. 99. ❑ N/A

reis5 – Have you been vaccinated against hepatitis A? If so, was the last time more or less than 10 years ago?

1. ❑ yes, I think that the last time was less than 10 years ago
2. ❑ yes, I think the last time was more than 10 years ago
3. ❑ yes, but I don’t remember when
4. ❑ I don’t know if I’ve been vaccinated
5. ❑ no

97. ❑ d.k. 98. ❑ n.a. 99. ❑ N/A

ACCULTURATION

*Int. Only ask the following questions (acc1 to acc8) if the respondent was not born in the Netherlands and/or if one of the parents was not born in the Netherlands*

Acc1 – Do you speak Dutch with your… [INT. WRITE ANSWER IN TABLE 4]

a. ❑ partner?

b. ❑ parents?

c. ❑ brothers and sisters?

d. ❑ children?

e. ❑ friends/acquaintances?

TABLE 4 Acculturation

| Do you speak Dutch with your… | yes, often/always | yes, sometimes | no, never | N/A |  |
| --- | --- | --- | --- | --- | --- |
| a. partner? | 1. ❑ | 2. ❑ | 3. ❑ | 4. ❑ |  d.k.  n.a. |
| b. parents? | 1. ❑ | 2. ❑ | 3. ❑ | 4. ❑ |  d.k.  n.a. |
| c. brothers and sisters? | 1. ❑ | 2. ❑ | 3. ❑ | 4. ❑ |  d.k.  n.a. |
| d. children? | 1. ❑ | 2. ❑ | 3. ❑ | 4. ❑ |  d.k.  n.a. |
| e. friends/acquaintances? | 1. ❑ | 2. ❑ | 3. ❑ | 4. ❑ |  d.k.  n.a. |

acc2 – How often do you watch Dutch film, television or video programs?

1. ❑ never
2. ❑ once a week or less
3. ❑ 1-3 times per week
4. ❑ 4-6 times per week
5. ❑ daily

97. ❑ d.k. 98. ❑ n.a. 99. ❑ N/A

acc3 – Which daily newspapers do you read at least once a week? [INT. SEVERAL ANSWERS POSSIBLE]

1. ❑ Dutch
2. ❑ Turkish
3. ❑ Moroccan
4. ❑ Other, namely………………………………………………………………………………………………
5. ❑ N.A /cannot read/illiterate

97. ❑ d.k. 98. ❑ n.a.

acc4 – Where do you usually buy your foodstuffs? At a foreign shop (such as a Turkish or Moroccan shop, or an Asian shop (toko)), a Dutch shop or at both?

1. ❑ foreign
2. ❑ Dutch
3. ❑ both
4. ❑ other/ neither

97. ❑ d.k. 98. ❑ n.a. 99. ❑ N/A

acc5 – Do you sometimes associate with (white) Dutch people in your leisure time?

1. ❑ yes, often 🡪 go to **acc6**
2. ❑ yes, sometimes 🡪 go to **acc6**
3. ❑ no, never 🡪 go to **acc8**

97. ❑ d.k. 98. ❑ n.a. 99. ❑ N/A

acc6 – In your leisure time, do you have more contact with (white) Dutch people than with people from your own ethnic group?

1. ❑ more contact with (white) Dutch people
2. ❑ more contact with people from your 'own' ethnic group
3. ❑ just as much contact with both

97. ❑ d.k. 98. ❑ n.a. 99. ❑ N/A

acc7 – How many of your best friends are (white) Dutch people?

1. ❑ none
2. ❑ one
3. ❑ a few
4. ❑ most of them
5. ❑ all

97. ❑ d.k. 98. ❑ n.a. 99. ❑ N/A

acc8 – Please indicate to what extent you agree or disagree with the following statements? [INT. SHOW CARD 13 ]

|  | Totally agree | agree | neither agree nor disagree | Disagree | Totally disagree | 97. 98. 99. |
| --- | --- | --- | --- | --- | --- | --- |
| a. Only the wife should take Important decisions concerning the children. | 1. ❑ | 2. ❑ | 3. ❑ | 4. ❑ | 5. ❑ |  d.k.  n.a.  N/A. |
| b. In the end, decisions concerning large purchases can best be taken by the husband. | 1. ❑ | 2. ❑ | 3. ❑ | 4. ❑ | 5. ❑ |  d.k.  n.a.  N/A |
| c. The wife is the best person to be responsible for the household. | 1. ❑ | 2. ❑ | 3. ❑ | 4. ❑ | 5. ❑ |  d.k.  n.a.  N/A |
| d. The husband is the best person to be responsible for the finances. | 1. ❑ | 2. ❑ | 3. ❑ | 4. ❑ | 5. ❑ |  d.k.  n.a.  N/A |
| e. An education is more important for boys than for girls. | 1. ❑ | 2. ❑ | 3. ❑ | 4. ❑ | 5. ❑ |  d.k.  n.a.  N/A |
| f. A woman must stop working when she has a child. | 1. ❑ | 2. ❑ | 3. ❑ | 4. ❑ | 5. ❑ |  d.k.  n.a.  N/A |

GHQ and NESDA

tekst-nesd: The following questions are about how you have been lately.

ghq01 – Have you recently lost much sleep over worry? [INT. SHOW CARD 14]

1. ❑ Not at all
2. ❑ No more than usual
3. ❑ Rather more than usual
4. ❑ Much more than usual

97. ❑ d.k. 98. ❑ n.a. 99. ❑ N/A

ghq02 – Have you recently felt constantly under strain? [INT. SHOW CARD 14]

1. ❑ Not at all
2. ❑ No more than usual
3. ❑ Rather more than usual
4. ❑ Much more than usual

97. ❑ d.k. 98. ❑ n.a. 99. ❑ N/A

ghq03 – Have you recently been able to concentrate on what you are doing? [INT. SHOW CARD 15]

1. ❑ Better than usual
2. ❑ Same as usual
3. ❑ Less than usual
4. ❑ Much less than usual

97. ❑ d.k. 98. ❑ n.a. 99. ❑ N/A

ghq04 – Have you recently felt that you are playing a useful part in things? [INT. SHOW CARD 16]

1. ❑ More so than usual
2. ❑ Same as usual
3. ❑ Less so than usual
4. ❑ Much less than usual

97. ❑ d.k. 98. ❑ n.a. 99. ❑ N/A

ghq05 – Have you recently been able to face up to your problems? [INT. SHOW CARD 17]

1. ❑ More so than usual
2. ❑ Same as usual
3. ❑ Less so than usual
4. ❑ Much less than usual

97. ❑ d.k. 98. ❑ n.a. 99. ❑ N/A

ghq06 – Have you recently felt capable of making decisions about things? [INT. SHOW CARD 18]

1. ❑ More so than usual
2. ❑ Same as usual
3. ❑ Less so than usual
4. ❑ Much less than usual

97. ❑ d.k. 98. ❑ n.a. 99. ❑ N/A

ghq07 – Have you recently felt you coundn’t overcome your difficulties? [INT. SHOW CARD 19]

1. ❑ Not at all
2. ❑ No more than usual
3. ❑ Rather more than usual
4. ❑ Much more than usual

97. ❑ d.k. 98. ❑ n.a. 99. ❑ N/A

ghq08 – Have you recently been feeling reasonably happy, all things considered? [INT. SHOW CARD 20]

1. ❑ More so than usual
2. ❑ Same as usual
3. ❑ Less so than usual
4. ❑ Much less than usual

97. ❑ d.k. 98. ❑ n.a. 99. ❑ N/A

ghq09 – Have you recently been able to enjoy your normal day to day activities? [INT. SHOW CARD 21]

1. ❑ More so than usual
2. ❑ Same as usual
3. ❑ Less so than usual
4. ❑ Much less than usual

97. ❑ d.k. 98. ❑ n.a. 99. ❑ N/A

ghq10 – Have you recently been feeling unhappy or depressed? [INT. SHOW CARD 22]

1. ❑ Not at all
2. ❑ No more than usual
3. ❑ Rather more than usual
4. ❑ Much more than usual

97. ❑ d.k. 98. ❑ n.a. 99. ❑ N/A

ghq11 – Have you recently been losing confidence in yourself? [INT. SHOW CARD 22]

1. ❑ Not at all
2. ❑ No more than usual
3. ❑ Rather more than usual
4. ❑ Much more than usual

97. ❑ d.k. 98. ❑ n.a. 99. ❑ N/A

ghq12 – Have you recently been thinking of yourself as a worthless person? [INT. SHOW CARD 22]

1. ❑ Not at all
2. ❑ No more than usual
3. ❑ Rather more than usual
4. ❑ Much more than usual

97. ❑ d.k. 98. ❑ n.a. 99. ❑ N/A

In the last 4 weeks, ....

| [INT. SHOW CARD 23] | all of the time | most of the time | some of the time | a little of the time | none of the time | 97. 98. 99. |
| --- | --- | --- | --- | --- | --- | --- |
| nesd1 - how often did you feel tired out for no good reasons? | 1. ❑ | 2. ❑ | 4. ❑ | 5. ❑ | 6. ❑ | ❑ d.k. ❑ n.a. ❑ N/A |
| nesd2 - how often did you feel nervous? | 1. ❑ | 2. ❑ | 4. ❑ | 5. ❑ | 6. ❑ | ❑ d.k. ❑ n.a. ❑ N/A |
| nesd3 - how often did you feel so nervouw that nothing could calm you down? | 1. ❑ | 2. ❑ | 4. ❑ | 5. ❑ | 6. ❑ | ❑ d.k. ❑ n.a. ❑ N/A |
| nesd4 - how often did you feel hopeless? | 1. ❑ | 2. ❑ | 4. ❑ | 5. ❑ | 6. ❑ | ❑ d.k. ❑ n.a. ❑ N/A |
| nesd5 - how often did you feel restless or fidgety? | 1. ❑ | 2. ❑ | 4. ❑ | 5. ❑ | 6. ❑ | ❑ d.k. ❑ n.a. ❑ N/A |
| nesd6 - how often did you feel so restless that you could not sit still? | 1. ❑ | 2. ❑ | 4. ❑ | 5. ❑ | 6. ❑ | ❑ d.k. ❑ n.a. ❑ N/A |
| nesd7 - how often did you feel depressed? | 1. ❑ | 2. ❑ | 4. ❑ | 5. ❑ | 6. ❑ | ❑ d.k. ❑ n.a. ❑ N/A |
| nesd8 - how often did you feel that everything was an effort? | 1. ❑ | 2. ❑ | 4. ❑ | 5. ❑ | 6. ❑ | ❑ d.k. ❑ n.a. ❑ N/A |
| nesd9 - how often did you feel so sad that nothing could cheer you up? | 1. ❑ | 2. ❑ | 4. ❑ | 5. ❑ | 6. ❑ | ❑ d.k. ❑ n.a. ❑ N/A |
| nesd10 – how often did you feel worthless? | 1. ❑ | 2. ❑ | 4. ❑ | 5. ❑ | 6. ❑ | ❑ d.k. ❑ n.a. ❑ N/A |

nesd11 – Did you suffer a spell or attack in the past month, during which you suddenly felt frightened, anxious or suddenly had many physical symptoms?

1. ❑ yes
2. ❑ no

97. ❑ d.k. 98. ❑ n.a. 99. ❑ N/A

nesd12 – Did you feel a strong fear to leave the house alone in the past month, to be in a crowd, to stand in line, or to travel by bus or by train?

1. ❑ yes
2. ❑ no

97. ❑ d.k. 98. ❑ n.a. 99. ❑ N/A

nesd13 – Did you feel a strong fear to do things in front of others in the past month, such as speaking, eating, or writing?

1. ❑ yes
2. ❑ no

97. ❑ d.k. 98. ❑ n.a. 99. ❑ N/A

nesd14 – Did you go through a period in the past month during which you felt worried, nervous, tense, or anxious most of the time?

1. ❑ yes
2. ❑ no

97. ❑ d.k. 98. ❑ n.a. 99. ❑ N/A

INFECTIOUS DISEASES

tekst-inf: Infectious diseases are a big problem in Amsterdam. For people themselves they can be annoying and even have serious consequences. We would like to know how often infectious diseases, such as those transmitted through blood contact or sex, occur. That’s why we would like to ask you some questions. Once again I would like to stress that the information shall be treated confidentially.

inf1 - In general you feel attracted to …. [INT. HELP IF NECESSARY]

1. ❑ Only women
2. ❑ Mainly women
3. ❑ Women as well as to men
4. ❑ Mainly men
5. ❑ Only men

97. ❑ d.k. 98. ❑ n.a. 99. ❑ N/A

inf2 – Have you ever been tested for HIV?

1. ❑ Yes, once 🡪 go to **inf3**
2. ❑ Yes, more often than once 🡪 go to **inf3**
3. ❑ No 🡪 go to the next block, **tekst-seks**

97. ❑ d.k. 98. ❑ n.a. 99. ❑ N/A

inf3 –When was the last time?

1. ❑ 2004
2. ❑ 2003
3. ❑ 2002
4. ❑ 2001
5. ❑ 2000
6. ❑ 1980-2000

97. ❑ d.k. 98. ❑ n.a. 99. ❑ N/A

SEXUAL CONTACT

tekst-seks: what follows are a number of questions about your sexual contacts. It is conceivable that you experience these questions as burdensome or annoying. Of course, you may at all times refuse to answer a question. Is it okay with you if we ask you these questions, would you like to fill them in yourself, or would you rather skip these questions?

1. ❑ continue 🡪 go to **seks1**
2. ❑ fill in myself
3. ❑ skip 🡪 go to the next block, **tekst-hep**

**INT. All questions about ‘sex’ are related to the past year.**

seks1 – Have you had one or more partners in the past year? (including steady partner or spouse and casual partners)

1. ❑ no 🡪 go to the next block, **tekst-hep**
2. ❑ yes, but no casual partners 🡪 go to **seks2**
3. ❑ yes, only casual partners 🡪 go to **seks2**
4. ❑ yes, steady partner as well as casual partners 🡪 go to **seks2**

97. ❑ d.k. 98. ❑ n.a. 99. ❑ N/A

*seks2 – How many partners have you had in the past year?* [several answers are possible and also think of your steady partner or spouse]

| 1. |  |  | man/men | (fill in the number) | |
| --- | --- | --- | --- | --- | --- |
| 2. |  |  | woman/women | (fill in the number) | |
| 97. ❑ d.k. 98. ❑ n.a. 99. ❑ N/A | | | | |  |

seks3 – Have you had sex in the past year (this includes anal and/or vaginal sex)?

1. ❑ yes 🡪 go to **seks4**
2. ❑ no 🡪 go to **tekst-hep**

97. ❑ d.k. 98. ❑ n.a. 99. ❑ N/A

seks4 – Have you had vaginal sex (sexual intercourse) with your partner(s) last year?

1. ❑ no, no vaginal sex with partners
2. ❑ yes, always with a condom
3. ❑ yes, sometimes with and sometimes without a condom
4. ❑ yes, never with a condom

97. ❑ d.k. 98. ❑ n.a. 99. ❑ N/A

*IN THE EVENT OF MALE PARTNER(S) (SEE QUESTION seks2)*

seks5 – Did you have anal sex with male partners in the past year?

1. ❑ no
2. ❑ yes, always with a condom
3. ❑ yes, sometimes with and sometimes without a condom
4. ❑ yes, never with a condom

97. ❑ d.k. 98. ❑ n.a. 99. ❑ N/A

seks6 – Have you had sex with people from a different population group than yours in the Netherlands in the past year?

1. ❑ yes 🡪 go to **seks7**
2. ❑ no 🡪 go to the next block, **tekst-hep**

97. ❑ d.k. 98. ❑ n.a. 99. ❑ N/A

seks7 – To which population group did these partners mostly belong (check no more than 3)?

1. ❑ Dutch

2. ❑ Surinamese

3. ❑ Creole Surinamese

4. ❑ Hindustani Surinamese

5. ❑ Chinese Surinamese

6. ❑ Javanese Surinamese

7. ❑ Antillian

8. ❑ Aruban

9. ❑ Moroccan

10. ❑ Berber

11. ❑ Turkish

12. ❑ Kurdish

13. ❑ other, namely …………………………………………………

97. ❑ d.k. 98. ❑ n.a. 99. ❑ N/A

HEPATITIS RISK FACTORS

tekst-hep: The following questions are asked to see whether you have run any risk of blood-transmittable infections, particularly hepatitis virus infections. Please indicate if you have **ever** experienced the following.

|  | **A** | **B** |
| --- | --- | --- |
| Have you… |  | *If the respondent has ever had this* ***outside the Netherlands*** *(“yes abroad and/or in the Netherlands” in column A):*  In which country or countries was this? |
| hep1 - …ever had injections? | 1. ❑ yes, in the Netherlands 2. ❑ yes, abroad and/or in the Netherlands 3. ❑ no     97. ❑ d.k.  98. ❑ n.a.  99. ❑ N/A | 1. ❑ Surinam  2. ❑ Netherlands Antilles  3. ❑ Aruba  4. ❑ Turkey  5. ❑ Morocco  6. ❑ Africa, namely………………………………..  7. ❑ Balkan  8. ❑ Soviet Union  9. ❑ Asia  10. ❑ other, namely………………………………… |
| hep2 - …ever had an operation? | 1. ❑ yes, in the Netherlands 2. ❑ yes, abroad and/or in the Netherlands 3. ❑ no     97. ❑ d.k.  98. ❑ n.a.  99. ❑ N/A | 1. ❑ Surinam  2. ❑ Netherlands Antilles  3. ❑ Aruba  4. ❑ Turkey  5. ❑ Morocco  6. ❑ Africa, namely………………………………..  7. ❑ Balkan  8. ❑ Soviet Union  9. ❑ Asia  10. ❑ other, namely………………………………… |
| hep3 - …ever had a tattoo? | 1. ❑ yes, in the Netherlands 2. ❑ yes, abroad and/or in the Netherlands 3. ❑ no     97. ❑ d.k.  98. ❑ n.a.  99. ❑ N/A | 1. ❑ Surinam  2. ❑ Netherlands Antilles  3. ❑ Aruba  4. ❑ Turkey  5. ❑ Morocco  6. ❑ Africa, namely………………………………..  7. ❑ Balkan  8. ❑ Soviet Union  9. ❑ Asia  10. ❑ other, namely………………………………… |
| hep4 - …ever had a blood transfusion? | 1. ❑ yes, in the Netherlands 2. ❑ yes, abroad and/or in the Netherlands 3. ❑ no     97. ❑ d.k.  98. ❑ n.a.  99. ❑ N/A | 1. ❑ Surinam  2. ❑ Netherlands Antilles  3. ❑ Aruba  4. ❑ Turkey  5. ❑ Morocco  6. ❑ Africa, namely………………………………..  7. ❑ Balkan  8. ❑ Soviet Union  9. ❑ Asia  10. ❑ other, namely………………………………… |

###### EDUCATION

*Int. Was the respondent born in the Netherlands 🡪 go to opl6*

*opl1-op56 are only to be asked if the resp. was not born in the Netherlands.*

Opl1 – Were you educated at a school outside the Netherlands?

1. ❑ yes 🡪 go to **opl2**
2. ❑ no 🡪 go to **opl5**

97. ❑ d.k. 98. ❑ n.a. 99. ❑ N/A

Opl2 - In what country was this?

1. ❑ Surinam 🡪 go to **opl3a**
2. ❑ Netherlands Antilles/Aruba 🡪 go to **opl3a**
3. ❑ Morocco 🡪 go to **opl3b**
4. ❑ Turkey 🡪 go to **opl3c**
5. ❑ elsewhere 🡪 go to **opl3d**

97. ❑ d.k. 98. ❑ n.a. 99. ❑ N/A

[INT. SHOW CARD 24]

opl3a – Using this card, please indicate what the highest level of education is that you have attained **in Surinam/the Netherlands Antilles/Aruba**.

1. ❑ elementary school/primary school not completed
2. ❑ elementary school /primary school completed
3. ❑ LBO/VBO (domestic science, trade, vocational, vocational technical school or an internal company training course) and accelerated upper secondary vocational education (MBO-kort)
4. ❑ lower general secondary education (MAVO), higher elementary education (MULO), advanced elementary education ( ULO)
5. ❑ upper secondary vocational education (long) (MBO‑lang), vocational training (BOL), vocational guidance (BBL)
6. ❑ upper general secondary education (HAVO), pre-university education (VWO), secondary modern school (HBS), girls’ secondary school (MMS)
7. ❑ higher professional education (HBO) /poly-technic
8. ❑ higher education (WO), university
9. ❑ other, namely__________________________________________________

97. ❑ d.k. 98. ❑ n.a. 99. ❑ N/A

IF R HASN’T FINISHED PRIMARY SCHOOL 🡪 go to **opl4**

OTHER 🡪 go to **opl5**

[INT. SHOW CARD 25]

opl3b – Using this card, please indicate what the highest level of education is that you have attained in **Morocco**.

1. ❑ *enseignement primaire* (primary education) not completed
2. ❑ *enseignement primaire* (primary education) completed
3. ❑ *enseignement secondaire premiere cycle* (secondary education 1st stage)
4. ❑ *enseignement secondaire seconde cycle* (secondary education 2nd stage)
5. ❑ education for a *diplôme de technicien* (technical diploma)
6. ❑ *enseignement supérieure* (pre-university education)
7. ❑ other, namely__________________________________________________

97. ❑ d.k. 98. ❑ n.a. 99. ❑ N/A

IF R HASN’T FINISHED PRIMARY SCHOOL 🡪 go to **opl4**

OTHER 🡪 go to **opl5**

[INT. SHOW CARD 26]

opl3c – Using this card, please indicate what the highest level of education is that you have attained in **Turkey**.

1. ❑ *Ilkokul* (elementary/primary school) not completed
2. ❑ *Ilkokul* (elementary/primary school) completed
3. ❑ *Sanat Okulu* (domestic science, trade, vocational, vocational technical school or an internal company training course) and accelerated upper secondary vocational education)
4. ❑ *Orta okul*
5. ❑ *Imam hatip okulu* (school for religious leaders)
6. ❑ *Lise* (lyceum)
7. ❑ *Ticaret Lisesi* (commercial lyceum)
8. ❑ higher education, university
9. ❑ other, namely__________________________________________________

97. ❑ d.k. 98. ❑ n.a. 99. ❑ N/A

IF R HASN’T FINISHED PRIMARY SCHOOL 🡪 go to **opl4**

OTHER 🡪 go to **opl5**

[INT. SHOW CARD 27]

opl3d – Using this card, please indicate what the highest level of education is that you have attained in **that country**.

1. ❑ elementary/primary school not completed
2. ❑ elementary/primary school completed
3. ❑ secondary school (vocational, lower secondary level)
4. ❑ secondary school (upper secondary vocational, higher general secondary, pre-university level)
5. ❑ secondary school, level unknown
6. ❑ higher professional education/poly-technic
7. ❑ university

97. ❑ d.k. 98. ❑ n.a. 99. ❑ N/A

*[INT. Only ask opl5 if the resp. has only had an elementary education.]*

Opl4 – How many years of elementary school did you follow?

|  |  | years | 97. ❑ d.k. 98. ❑ n.a. 99. ❑ N/A |
| --- | --- | --- | --- |

Opl5 – Did you go to school in the Netherlands?

1. ❑ Yes 🡪 go to **opl6**
2. ❑ No 🡪 go to **opl8**

97. ❑ d.k. 98. ❑ n.a. 99. ❑ N/A

[INT. Ask opl7 also if opl6=2 and the resp. was born in the Netherlands.] [INT. SHOW CARD 28]

Opl6 – Would you please indicate on the basis of this card what the highest level of education is that you followed in the Netherlands?

1. ❑ geen opleiding (lager onderwijs: niet afgemaakt 🡪 go to **opl7**
2. ❑ lager onderwijs (basisschool, speciaal basisonderwijs) 🡪 go to **opl8**
3. ❑ lager of voorbereidend beroepsonderwijs (zoals LTS, LEAO, LHNO, VMBO) 🡪 go to **opl8**
4. ❑ middelbaar algemeen voortgezet onderwijs (zoals MAVO, (M)ULO, MBO-kort, VMBO-t) 🡪 go to **opl8**
5. ❑ middelbaar beroepsonderwijs en beroepsbegeleidend onderwijs (zoals MBO‑lang, MTS, MEAO, BOL, BBL, INAS) 🡪 go to **opl8**
6. ❑ hoger algemeen en voorbereidend wetenschappelijk onderwijs (zoals HAVO, VWO, Atheneum, Gymnasium, HBS, MMS) 🡪 go to **opl8**
7. ❑ hoger beroepsonderwijs (zoals HBO, HTS, HEAO, HBO-V, kandidaats wetenschappelijk onderwijs) 🡪 go to **opl8**
8. ❑ wetenschappelijk onderwijs (universiteit) 🡪 go to **opl8**
9. ❑ other, namely……………………………………………………….. 🡪 go to **opl8**

97. ❑ d.k. 98. ❑ n.a. 99. ❑ N/A

Opl7 – How many years of elementary education did you follow?

|  |  | years | 97. ❑ d.k. 98. ❑ n.a. 99. ❑ N/A |
| --- | --- | --- | --- |

[INT. SHOW CARD 29]

Opl8 – Would you please indicate on the basis of this card what your mother’s highest diploma is?

1. ❑ elementary/primary school not completed
2. ❑ elementary/primary school completed
3. ❑ secondary school (lower secondary vocational, lower secondary general level)
4. ❑ secondary school (upper secondary vocational, upper secondary general, pre-university level)
5. ❑ secondary school, level unknown
6. ❑ higher professional education/poly-technic
7. ❑ university

97. ❑ d.k. 98. ❑ n.a. 99. ❑ N/A

Opl9 – And what is the highest diploma gained by your father, according to this card?

1. ❑ elementary/primary school not completed
2. ❑ elementary/primary school completed
3. ❑ secondary school (lower secondary vocational, lower secondary general level)
4. ❑ secondary school (upper secondary vocational, upper secondary general, pre-university level)
5. ❑ secondary school, level unknown
6. ❑ higher professional education /poly-technic
7. ❑ university

97. ❑ d.k. 98. ❑ n.a. 99. ❑ N/A

###### EMPLOYMENT SITUATION

[INT. SHOW CARD 30]

werk1 – Please look at this card. Which situation is most applicable to you? [INT.: only 1 answer possible]

1. ❑ I do paid work, 32 hours or more per week
2. ❑ I do paid work, 20 hours or more, but less than 32 hours per week
3. ❑ I do paid work, 12 hours or more, but less than 20 hours per week
4. ❑ I do paid work, less than 12 hours per week
5. ❑ I have retired (early) (AOW, VUT, FPU)
6. ❑ I am unemployed/looking for a job (registered at the Employment Exchange)
7. ❑ I am unfit for work (WAO, AAW, WAZ, WAJONG)
8. ❑ I am on national assistance
9. ❑ I am a full-time homemaker
10. ❑ I am a student

97. ❑ d.k. 98. ❑ n.a. 99. ❑ N/A

kost1 – Are you the main breadwinner? [INT.: the main breadwinner is the person bringing in the highest income in the household]

1. ❑ yes 🡪 go to **verz1**
2. ❑ no 🡪 go to **kost2**

97. ❑ d.k. 98. ❑ n.a. 99. ❑ N/A

*[Only ask kost2 if the resp. is not the main breadwinner.]*

[INT. SHOW CARD 30]

kost2 – I still have a few questions about the main breadwinner in this household. Can you indicate with the aid of this card what his or her primary occupation is, or to what group he or she belongs? [INT.: only 1 answer possible]

1. ❑ I do paid work 32 hours or more per week
2. ❑ I do paid work 20 hours or more, but less than 32 hours per week
3. ❑ I do paid work 12 uur or more but less than 20 hours per week
4. ❑ I do paid work less than 12 hours per week
5. ❑ I have retired (early) (AOW, VUT, FPU)
6. ❑ I am unemployed/ looking for a job (registered at the Employment Exchange)
7. ❑ I am unfit for work (WAO, AAW, WAZ, WAJONG)
8. ❑ I am on national assistance
9. ❑ I am a full-time homemaker
10. ❑ I am a student

97. ❑ d.k. 98. ❑ n.a. 99. ❑ N/A

verz1 – How are you ensured for medical expenses?

1. ❑ AGIS national health insurance fund (formerly ZAO)
2. ❑ another national health insurance fund
3. ❑ civil servants insurance (e.g. IZA, IZR, DGVP)
4. ❑ private health insurance
5. ❑ not insured

97. ❑ d.k. 98. ❑ n.a. 99. ❑ N/A

INCOME

tekst18-ink: There may be a connection between sickness and health and the income you can spend freely. Therefore, several questions follow below about the level of your income.

[INT. SHOW CARD 31]

Ink1 – What is the aggregate total NET MONTHLY INCOME at the disposal of your household? (this is without the income of children under 18, without holiday allowance, child benefit and side income)

1. ❑ € 700,- (ƒ1.543,-) or less
2. ❑ € 701,- (ƒ 1.544,-) to € 1.000,- (ƒ 2.204,-)
3. ❑ € 1.001,- (ƒ 2.206,-) to € 1.350,- (ƒ 2.975,-)
4. ❑ € 1.351,- (ƒ 2.997,-) to € 2.050,- (ƒ 4.518,-)
5. ❑ € 2.051,- (ƒ 4.519,-) to € 3.200,- (ƒ 7.052,-)
6. ❑ more than € 3.201,- (ƒ 7.054,-)
7. ❑ won’t say

97. ❑ d.k. 98. ❑ n.a. 99. ❑ N/A

ink2 – How many incomes are involved here (exclusive of that of any children under 18)?

1. ❑ one income
2. ❑ two incomes
3. ❑ more than two incomes

97. ❑ d.k. 98. ❑ n.a. 99. ❑ N/A

ink3 – Can you indicate the income components that comprise the net income of your household? [INT. MORE THAN ONE ANSWER POSSIBLE].

1. ❑ wage, salary (also from jobs for benefit claimants (Melkert jobs) etc.)
2. ❑ old age pension (AOW), surviving dependants pension (ANW)
3. ❑ pension, early retirement scheme (VUT)
4. ❑ State means-tested unemployment scheme (RWW), benefit
5. ❑ scholarship, parents’ contribution
6. ❑ disablement benefit (WAO)/disablement pension (AAW)
7. ❑ (non-)working jobseekers scheme ((N)WW)
8. ❑ other benefit scheme
9. ❑ own business, practice
10. ❑ own capital, annuity
11. ❑ other, namely…………………………………………………………………………………………………………………

97. ❑ d.k. 98. ❑ n.a. 99. ❑ N/A

ink4 – What is the financial situation of your household at present: do you have to go into debt, use savings, do you just get by, have a bit of money left or a lot of money left?

1. ❑ have to go into debt
2. ❑ have to use savings
3. ❑ just get by
4. ❑ have a bit of money left
5. ❑ have a lot of money left

97. ❑ d.k. 98. ❑ n.a. 99. ❑ N/A

IMPROVEMENT OF HEALTH

Text19 – At the end of this interview, we would like to ask you if you yourself have any ideas about how you can improve your health.

gzhb1 – Which changes in your environment would you wish MOST to improve your health? Please mention only the most important. [INT.: DO NOT READ OUT! Find the right category and enter *one* code!]

1. ❑ more facilities (a community centre or sports centre)
2. ❑ more social support from family and friends
3. ❑ more professional support (accessibility, quantity, quality)
4. ❑ a better environment (less noise, stench, nuisance from neighbours)
5. ❑ more rules and prohibitions (less aggression on the streets, more police, more safety)
6. ❑ something else, namely……………………………………………………………………………………………...…………
   ……………………………………………………………………………………………………………………………………..…..
7. ❑ nothing

97. ❑ d.k. 98. ❑ n.a. 99. ❑ N/A

gzhb2 – What do you think you yourself can do to improve your health? Please mention only the most important.

[INT.: DO NOT READ OUT! Find the right category and enter *one* code!]

1. ❑ drink less
2. ❑ smoke less
3. ❑ take more physical exercise
4. ❑ eat healthier
5. ❑ take things a bit easier
6. ❑ solve personal problems
7. ❑ have more contacts with other people
8. ❑ improve medical behaviour (see my doctor, take less medication)
9. ❑ something else, namely……………………………………………………………………………………………………….
   …………………………………………………………………………………………………………………………………………
10. ❑ nothing 🡪 go to **aqui1**

97. ❑ d.k. 98. ❑ n.a. 99. ❑ N/A

gzhb3 – What makes it hard for you to change this? Please mention only the most important obstacle.

[INT.: DO NOT READ OUT! Find the right category and enter *one* code!]

1. ❑ lack of knowledge or information
2. ❑ lack of money
3. ❑ lack of willpower
4. ❑ insufficient support from friends or family
5. ❑ insufficient professional support
6. ❑ bad facilities or services
7. ❑ something else, namely………………………………………………………………………………………………………
   …………………………………………………………………………………………………………………………………………
8. ❑ nothing

97. ❑ d.k. 98. ❑ n.a. 99. ❑ N/A

aqui1 – Have you heard, seen or read anything about the campaign "heartbeat Amsterdam, healthy together"?

1. ❑ yes 🡪 go to **aqui2**
2. ❑ no 🡪 go to **verv1**

97. ❑ d.k. 98. ❑ n.a. 99. ❑ N/A

aqui2 – Where did you hear, see or read about this?

1. ❑ folder
2. ❑ indirectly, from friends/acquaintances
3. ❑ general practitioner or other specialist
4. ❑ GG&GD
5. ❑ television
6. ❑ radio
7. ❑ newspaper/ magazine
8. ❑ somewhere else

97. ❑ d.k. 98. ❑ n.a. 99. ❑ N/A

verv1 – We have almost reached the end of the questionnaire. To conclude, I would like to ask you if the GG&GD may approach you again for a GG&GD follow-up survey? Of course, you can always decide whether or not you want to take part at the time when you are invited to do so.

1. ❑ yes
2. ❑ no

tekst-eind: We have now come to the end of the questionnaire part of this survey. The physical examination part now follows. Before starting this, I would first like to go through the following with you.

INT. PROCEED WITH ‘PERMISSION STATEMENT’

PART TO BE FILLED IN BY INTERVIEWER

INT. Below, fill in the time when you end this interview

| time |  |  | : |  |  |
| --- | --- | --- | --- | --- | --- |

enq1 – The interview was held with…

1. ❑ respondent only 🡪 enq3
2. ❑ others present, not annoying 🡪 enq2
3. ❑ others present, annoying 🡪 enq2
4. ❑ other annoyances 🡪 enq3

enq2 – Who was also present during the interview?

1. ❑ …………………………………………………………………

enq3 – The respondent was…

1. ❑ very cooperative
2. ❑ simply cooperative
3. ❑ not very cooperative
4. ❑ no opinion

enq4 - Respondent seemed to consider the interview…

1. ❑ threatening
2. ❑ not threatening
3. ❑ no opinion

enq5a – Which questions did the respondent consider threatening (3 questions at most)?

Question number:



| 1. ❑ |  |
| --- | --- |
| 1. ❑ |  |
| 1. ❑ |  |
| 1. ❑ | none |
